# Efficacy and safety of jinlida granule in the treatment of diabetic kidney disease: a systematic review and meta-analysis of randomized controlled trials

**DOI:** 10.3389/fphar.2026.1761454

**Published:** 2026-06-03

**Authors:** Qi Yong, Yunxi Xu, Peiying Zhang, Hua Bai, Chao Xu, Hejiang Ye

**Affiliations:** 1 Clinical Medical College, Chengdu University of Traditional Chinese Medicine, Chengdu, China; 2 Department of Ophthalmology, Hospital of Chengdu University of Traditional Chinese Medicine, Chengdu, China

**Keywords:** diabetic kidney disease, jinlida granule, meta‐analysis, randomized controlled trial, renal protection, traditional Chinese medicine

## Abstract

**Background:**

Diabetic kidney disease (DKD), a major microvascular complication of diabetes, represents the leading cause of end-stage renal disease globally. Jinlida Granule (JLD), a multi-botanical Traditional Chinese Medicine preparation, has shown potential in type 2 diabetes management; however, a comprehensive systematic evaluation of its efficacy and safety specifically for DKD is lacking.

**Objective:**

To systematically evaluate the efficacy and safety of JLD for DKD through a meta-analysis.

**Methods:**

We conducted a systematic search of Chinese and English databases and clinical trial registries for randomized controlled trials (RCTs) on JLD for DKD from inception to 9 October 2025. Two researchers independently performed literature screening, data extraction, and quality assessment using the Cochrane Risk of Bias (RoB) 2 tool. Meta-analysis was conducted with a random-effects model using STATA 15.0 and Review Manager 5.4. Subgroup and sensitivity analyses were performed.

**Results:**

Twelve RCTs involving 1,341 patients were included. JLD was associated with significant reductions in serum creatinine (MD −30.54 μmol/L, 95% CI −37.61 to −23.47; I^2^ = 94%), blood urea nitrogen (MD −0.95 mmol/L, 95% CI −1.29 to −0.60; I^2^ = 79%), urinary albumin excretion rate (MD −27.09 mg/24 h, 95% CI −29.16 to −25.02; I^2^ = 94%), and HbA1c (MD −0.86%, 95% CI −1.23% to −0.49%; I^2^ = 83%). Improvements were also observed in lipid profiles and inflammatory markers. Subgroup analyses suggested greater benefit in early-stage DKD and with specific combination regimens. Adverse events did not differ significantly between groups (RR 0.86, 95% CI 0.50–1.48). Substantial heterogeneity was noted.

**Conclusion:**

Current evidence suggests that adjunctive JLD may provide multifaceted clinical benefits for DKD patients, with a comparable safety profile. However, these findings should be interpreted cautiously given the moderate to high risk of bias and considerable heterogeneity. Well-designed, large-scale RCTs are required.

**Systematic Review Registration:**

https://www.crd.york.ac.uk/PROSPERO/view/CRD420251178318, identifier [CRD420251178318].

## 1 Introduction

Diabetic kidney disease (DKD) is one of the most severe and common microvascular complications of diabetes and has emerged as the primary cause of end-stage renal disease (ESRD) worldwide, posing a significant public health burden (Sun et al., 2022). According to the latest International Diabetes Federation data, approximately 537 million adults have diabetes, a number projected to rise to 783 million by 2045 (Sun et al., 2022). Approximately 30%–40% of diabetic patients progress to DKD (Alicic et al., 2021), whose high treatment costs and substantial disease burden present severe challenges to both individuals and healthcare systems.

The pathophysiology of DKD is complex and multidimensional, driven by more than just chronic hyperglycemia (DeFronzo et al., 2021). It involves hemodynamic changes, glycolipid metabolic disorders, oxidative stress, a chronic micro-inflammatory state, and the interplay of various programmed cell death pathways (e.g., apoptosis, pyroptosis), ultimately leading to progressive glomerulosclerosis and tubulointerstitial fibrosis (Ansari et al., 2025; Liu et al., 2024). Notably, recent studies have confirmed that mitochondrial dysfunction is a critical nexus connecting metabolic disorders to renal cell injury and “sterile inflammation” (Hu et al., 2025; Mitrofanova et al., 2022).

Recent years have witnessed landmark advances in DKD treatment strategies. The traditional standard-of-care comprises strict glycemic control and blood pressure management based on renin-angiotensin system (RAS) inhibitors. This paradigm has been revolutionized by novel agents such as sodium-glucose cotransporter-2 inhibitors (SGLT2i) and glucagon-like peptide-1 receptor agonists (GLP-1 RAs) (Johnson and Prasad-Reddy, 2024; Winiarska et al., 2021). These drugs, by virtue of their clear cardiorenal protective benefits independent of glucose-lowering effects, have transformed the treatment landscape and received strong endorsement in international guidelines. The 2024 Kidney Disease: Improving Global Outcomes (KDIGO) Clinical Practice Guideline strongly recommends SGLT2i for chronic kidney disease (CKD) patients with or without type 2 diabetes (Madero et al., 2025).

However, a significant gap exists between guideline recommendations and real-world clinical practice (Wanner et al., 2025). A real-world study based on a large United Kingdom primary care database revealed that the overall prescription rate of SGLT2i among eligible CKD patients was only 17.0%; more alarmingly, this figure dropped to below 0.1% in patients without T2D (Forbes et al., 2024). This stark “guideline-practice gap,” coupled with the fact that the long-term cardiorenal benefits and risks of widely used therapies (e.g., TZDs) in the DKD population are still based on low-certainty evidence (Natale et al., 2025) and the complex, multi-mechanism, multi-pathway pathophysiology of DKD itself (Daehn et al., 2023; DeFronzo et al., 2021), underscores the urgency and immense clinical value of actively exploring effective, safe, and accessible adjuvant treatment strategies to complement current standard care.

Within this context, Traditional Chinese Medicine (TCM), characterized by holistic regulation and multi-target intervention, offers a unique perspective and potential solutions for DKD prevention and treatment (Li et al., 2025). In TCM theory, DKD falls under the category of “Xiaoke Nephropathy,” with its core pathogenesis rooted in Qi and Yin deficiency, progressing to spleen and kidney deficiency, with pathological products like stasis, phlegm, and turbid toxins obstructing the kidney collaterals, forming the key pathogenesis of “toxin damaging the kidney collaterals” (Deng et al., 2020). Jinlida Granule (JLD) is a compound formulation developed based on the TCM “Treating from the Spleen” theory, approved in 2005 for T2D treatment. Composed of 17 botanical drugs, including Ginseng Radix et Rhizoma (Renshen), Polygonati Rhizoma (Huangjing), and Salviae Miltiorrhizae Radix et Rhizoma (Danshen), its composition follows the strict principles of monarch, minister, assistant, and guide, collectively achieving the effects of replenishing Qi, nourishing Yin, fortifying the spleen, and activating collaterals.

The development and application of JLD are deeply rooted in the theoretical evolution of traditional Chinese medicine (TCM) regarding “Xiaoke” (wasting-thirst disorder). Classical TCM theory posits that the core pathogenesis of “Xiaoke” involves spleen dysfunction leading to disturbances in fluid and nutrient metabolism. The ancient medical canon Yellow Emperor’s Inner Classic (Jing, 2011) states, “Those who are fond of fatty and sweet foods. Their internal heat becomes abundant, leading to wasting-thirst” (Classics, −200), highlighting the role of diet and internal heat. Modern TCM scholarship, particularly the “Collateral Disease (Luo-Bing) Theory” pioneered by Academician Wu Yiling, further developed the concept of “treating diabetes from the spleen” (Yiling, 2004). This theory holds that “spleen deficiency and collateral stasis” is the fundamental pathological change, where impaired spleen transportation leads to the accumulation of pathological products like dampness, phlegm, and stasis, obstructing the collaterals and ultimately causing microvascular complications such as diabetic kidney disease (DKD).

JLD’s formula is a modern embodiment of this “treating from the spleen” principle. It is designed to “fortify the spleen and promote fluid circulation” (Jian Pi Yun Jin), aiming to restore the spleen’s function of transporting and transforming nutrients and fluids (Standardization Project of TCM Terminology, 2025). The formula’s composition reflects sophisticated TCM compatibility principles. Panax ginseng (Renshen) and Polygonati Rhizoma (Huangjing) serve as the monarch drugs to replenish Qi and nourish Yin, addressing the root deficiency. A cohort of minister botanical drugs, including Rehmanniae Radix (Dihuang), Ophiopogonis Radix (Maidong), and Anemarrhenae Rhizoma (Zhimu), assist in nourishing Yin and clearing heat. Crucially, spleen-fortifying botanical drugs like Atractylodis Rhizoma (Cangzhu) and Poria (Fuling) are used to directly resolve dampness and strengthen transportation. This is combined with botanical drugs that clear heat and dampness (e.g., Coptidis Rhizoma (Huanglian), Sophorae Flavescentis Radix (Kushen)) and activate blood circulation to remove stasis (e.g., Salviae Miltiorrhizae Radix et Rhizoma (Danshen)), collectively preventing pathological factors from damaging the collaterals.

Therefore, JLD’s traditional therapeutic rationale for intervening in DKD is multidimensional: its Qi and Yin tonifying action corrects the underlying deficiency pattern; its spleen-fortifying and dampness-resolving action directly targets the core dysfunction of “spleen deficiency and damp encumbrance”; and its heat-clearing and blood-activating components address the accompanying stasis and toxins that obstruct the kidney collaterals. Clinical research conducted under the guidance of this collateral vessel theory has demonstrated that JLD, especially in combination with other collateral-activating botanical preparations, can significantly improve renal function indicators (e.g., urinary albumin) in patients with type 2 DKD, likely through mechanisms involving glucose-lowering, lipid regulation, and microcirculation improvement (Commission, 2020). This provides a coherent, theory-driven rationale for investigating JLD’s efficacy in mitigating the progression from metabolic disorder to renal microvascular injury.

Preliminary basic research has revealed its multi-target mechanisms: its bioactive metabolites may improve mitochondrial function and homeostasis by modulating the AMPK/PGC-1α pathway (Sun et al., 2025), exert antioxidant effects via activating the Nrf2 pathway (Tanase et al., 2022), and confer anti-inflammatory effects by inhibiting the NF-κB pathway, among others (Deng et al., 2024). These mechanisms precisely intervene in the core processes of DKD progression (Hu et al., 2025; Liu et al., 2024).

Although JLD is recommended as an adjuvant botanical therapy for diabetes in the “Chinese Guidelines for the Prevention and Treatment of Type 2 Diabetes” and has accumulated considerable clinical and basic research evidence, a systematic, comprehensive, and quantitative summary of evidence regarding its efficacy and safety specifically for the complication of diabetic kidney disease is lacking. Therefore, this study aims to employ a rigorous and standardized systematic review and meta-analysis approach to synthesize existing evidence from randomized controlled trials (RCTs) to comprehensively evaluate the efficacy and safety of JLD in treating DKD, thereby providing high-level evidence-based support for its potential role as a promising integrative adjuvant therapy bridging the current guideline-practice gap.

The comprehensive analysis of available evidence through systematic review and meta-analysis aims to provide high-level evidence-based support for JLD’s potential role in DKD management.

## 2 Materials and methods

This systematic review and meta-analysis was conducted and reported in accordance with the Preferred Reporting Items for Systematic Reviews and Meta-Analyses (PRISMA) guidelines. The review protocol was registered prospectively with PROSPERO (CRD420251178318).

### 2.1 Search strategy

We performed comprehensive searches in major Chinese and English electronic databases and clinical trial registries from their inception until 9 October 2025, to identify all relevant RCTs investigating Jinlida Granule for DKD. Chinese search terms included: “津力达颗粒”, “金力达颗粒”, “糖尿病肾病”, “糖尿病肾脏疾病”, “随机对照试验”, etc. English search terms included: “Jinlida Granule”, “Jinlida”, “Diabetic Nephropathies”, “Diabetic Kidney Disease”, “Randomized Controlled Trial”, etc. A combination of subject headings and free-text terms was used for retrieval, and search strategies were adjusted according to the characteristics of different databases. Additionally, reference lists of included studies were manually searched to supplement relevant literature.

### 2.2 Inclusion and exclusion criteria

#### 2.2.1 Inclusion criteria

- Study design: Randomized controlled trials, regardless of blinding.
- Participants: Patients clinically diagnosed with diabetic kidney disease, meeting international (e.g., 2010 American Diabetes Association criteria, WHO standards, or 2012 NKF-K/DOQI guidelines) or domestically recognized standards (e.g., “Diabetic Kidney Disease Integrated Diagnosis and Treatment Guidelines”, “Chinese Type 2 Diabetes Prevention and Treatment Guidelines”, etc.), without restriction on specific stages.
- Interventions: The experimental group received Jinlida Granule in addition to control group treatment, or used an integrated Chinese-biomedicine regimen with Jinlida Granule as the core.
- Comparisons: The control group received conventional biomedicine treatment, including but not limited to glucose-lowering, antihypertensive, lipid-regulating agents, and renin-angiotensin system inhibitors.
- Outcomes: Studies needed to report at least one of the following indicators: ① Renal function indicators (e.g., serum creatinine, blood urea nitrogen, urinary albumin excretion rate, 24-h urinary protein quantification); ② Glycemic metabolic indicators (e.g., fasting blood glucose, 2-h postprandial blood glucose, glycated hemoglobin); ③ Lipid indicators (e.g., triglycerides, total cholesterol); ④ Inflammatory indicators (e.g., interleukin-6, tumor necrosis factor-α, high-sensitivity C-reactive protein); ⑤ Safety indicators (adverse event incidence rate).

#### 2.2.2 Exclusion criteria

- Study design: Non-randomized controlled trial designs, such as reviews, case reports, animal experiments, *in vitro* studies; literature with incomplete data, unavailable full text, or duplicate publications.
- Participants: Patients with acute metabolic disorders (e.g., diabetic ketoacidosis or infection), severe liver impairment (ALT/AST greater than 2.5 times the upper limit of normal), severe renal impairment (serum creatinine greater than the upper limit of normal or eGFR ≤60 mL/min/1.73 m^2^), severe cardiovascular disease, or during pregnancy and lactation.
- Interventions: Experimental group interventions containing other Chinese botanical compounds or extracts that may affect efficacy evaluation.
- Comparisons: Control group interventions that are unclear or differ too much from the experimental group, preventing effective comparison.

### 2.3 Botanical nomenclature verification

In accordance with the nomenclature requirements for ethnopharmacological research, the botanical names of all herbal components contained in Jinlida Granule were verified. The scientific names (including authorities) and plant families were checked against the Medicinal Plant Names Services (MPNS) portal (http://mpns.kew.org). The verification was conducted on 2025-11–19. The full botanical composition is presented in Table 1 in the Results section.

**TABLE 1:** Composition of jinlida granule.

Chinese name	Latin name (full with authority)	Plant family	Part used	Weight ratio (%)
黄精	*Polygonatum kingianum* collett & hemsl	Asparagaceae	Rhizome	8.60
麦冬	*Ophiopogon japonicus* (thunb.) ker gawl	Asparagaceae	Tuber	8.60
山茱萸	*Cornus officinalis* siebold & zucc	Cornaceae	Fruit	8.60
葛根	*Pueraria montana* var. *lobata* (willd.) maesen & S.M.Almeida ex sanjappa & predeep	Fabaceae	Root	8.60
荔枝核	*Litchi chinensis* sonn	Sapindaceae	Seed	8.60
人参	*Panax ginseng* C.A.Mey	Araliaceae	Root and rhizome	6.49
地黄	*Rehmannia glutinosa* (gaertn.) libosch. ex fisch. & C.A.Mey	Orobanchaceae	Root	6.49
丹参	*Salvia miltiorrhiza* bunge	Lamiaceae	Root and rhizome	5.63
何首乌	*Reynoutria multiflora* (thunb.) moldenke	Polygonaceae	Prepared root	5.24
茯苓	*Wolfiporia cocos* (F.A. Wolf) ryvarden & gilb	Polyporaceae	Sclerotium	5.24
地骨皮	*Lycium chinense* mill	Solanaceae	Root bark	5.24
苍术	*Atractylodes lancea* (thunb.) DC.	Asteraceae	Rhizome (stir-baked)	4.30
知母	*Anemarrhena asphodeloides* bunge	Asparagaceae	Rhizome	4.30
苦参	*Sophora flavescens* aiton	Fabaceae	Root	3.52
佩兰	*Eupatorium fortunei* turcz	Asteraceae	Aerial part	3.52
黄连	*Coptis chinensis* franch	Ranunculaceae	Rhizome	3.52
淫羊藿	*Epimedium brevicornu* maxim	Berberidaceae	Leaf (processed)	3.52

Jinlida Granule is composed of 17 herbal ingredients with a total raw material input of approximately 2,842.9 g. The weight ratio was calculated based on the Chinese Pharmacopoeia (2020 Edition).

### 2.4 Literature screening and data extraction

This study imported search results into EndNote X9 software in bibliography form to establish a database. Literature screening and data extraction were carried out independently by two investigators (YQ and ZPY). Any discrepancies were resolved by consensus or, when necessary, by adjudication from a third senior researcher (XYX). First, titles and abstracts were read for initial screening to exclude obviously irrelevant literature; then, full texts of remaining literature were obtained for re-screening to determine finally included studies. A pre-designed data extraction form was used to extract information, including: first author, publication year, study design, sample size, patient baseline characteristics (e.g., age, gender, diabetes and diabetic kidney disease duration, stage), specific details of interventions (drug, dosage, usage, course), control measures, outcome indicator data (mean, standard deviation), etc.

### 2.5 Risk of bias assessment using Cochrane RoB 2 tool

The revised Cochrane risk-of-bias tool for randomized trials (RoB 2) was used to assess the methodological quality of the included studies. This assessment was completed independently by two researchers (YQ and ZPY). In case of disagreement, consensus was reached through discussion or consultation with a third researcher (XYX). Each study was evaluated across five domains: (1) bias arising from the randomization process, (2) bias due to deviations from intended interventions, (3) bias due to missing outcome data, (4) bias in measurement of the outcome, and (5) bias in selection of the reported result. The overall risk of bias was judged as “low risk of bias”, “some concerns”, or “high risk of bias” according to the RoB 2 algorithm.

### 2.6 Statistical analysis

STATA 15.0 and Review Manager 5.4 software were used for statistical analysis. For continuous variables, if measurement units were the same, mean difference was used; if measurement units differed or mean differences were large, standardized mean difference was used, both calculating their 95% confidence intervals. For dichotomous variables, risk ratio and its 95% confidence interval were calculated. The I^2^ statistic was used to assess heterogeneity between studies: I^2^ ≤ 25% indicated low heterogeneity, 25% < I^2^ ≤ 50% indicated moderate heterogeneity, I^2^ > 50% indicated high heterogeneity. A random-effects model was used for most pooled analyses (DerSimonian and Laird, 1986). The use of a random-effects model was pre-specified irrespective of the I^2^ statistic, given the inherent variability in participant characteristics, treatment protocols, and concomitant medications across the included studies. Subgroup analysis was preset according to disease stage (early-middle vs. late), baseline indicator levels, combination medication regimens, course duration, and other factors. Meta-regression was planned to explore the influence of treatment duration, baseline disease severity, and combination regimen type on the pooled effect estimates for outcomes with ten or more studies. However, given that all outcomes comprised fewer than ten studies, meta-regression would have been underpowered and was only conducted as an exploratory analysis for the primary outcome (Scr). The one-by-one exclusion method was used for sensitivity analysis to assess the robustness of combined results. For outcomes with seven or more included studies, potential publication bias was assessed by visual inspection of funnel plots, supplemented by Begg’s and Egger’s tests (Begg and Mazumdar, 1994; Egger et al., 1997). The limited statistical power of these tests when the number of studies is small was acknowledged. The certainty of evidence for each major outcome was assessed using the Grading of Recommendations Assessment, Development and Evaluation (GRADE) approach (Guyatt et al., 2008).

## 3 Results

### 3.1 Literature screening process and included study characteristics

**FIGURE 1:**
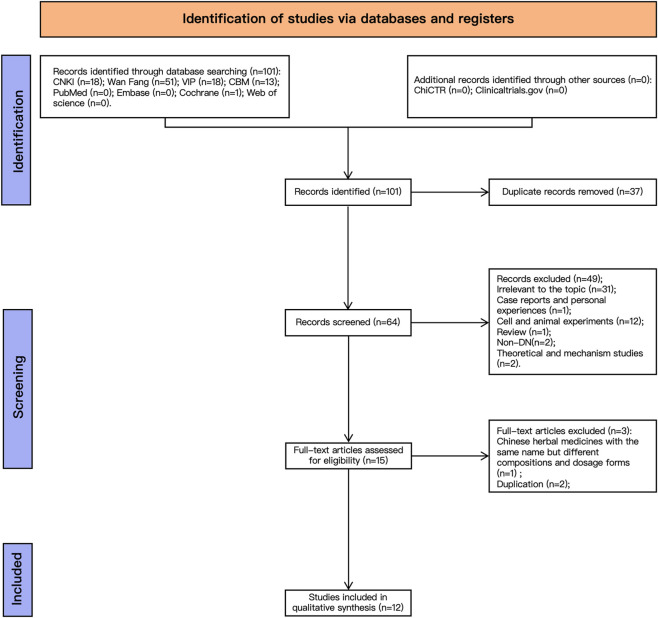
Flow diagram of the studies selection process. A total of 101 records were identified from databases (CNKI: 18, Wanfang: 51, VIP: 18, CBM: 13, Cochrane: 1). After excluding 49 records (irrelevant to the topic n = 31, case reports/personal experiences n = 1, cell/animal experiments n = 12, reviews n = 1, non‑diabetic nephropathy n = 2, theoretical/mechanistic studies n = 2), 15 full‑text articles were assessed for eligibility. Three articles were excluded (one due to different composition and dosage form, two due to duplication), and finally 12 randomized controlled trials were included.

We identified 101 records from databases (CNKI: 18, Wanfang: 51, VIP: 18, CBM: 13, Cochrane: 1). After excluding 49 records that were irrelevant to the topic (n = 31), case reports or personal experiences (n = 1), cell or animal experiments (n = 12), reviews (n = 1), non-diabetic nephropathy studies (n = 2), or theoretical and mechanistic studies (n = 2), 15 full-text articles were assessed for eligibility. After reading the full texts, 3 articles were excluded (one due to different composition and dosage form, two due to duplication), and finally 12 randomized controlled trials (Chen J. S. et al, 2019; Deng et al., 2020; Du et al., 2012; Liu et al., 2019; Liu and Zhang, 2024; Lou et al., 2018; Ren et al., 2019; Su, 2020; Su et al., 2017; Wang, 2023; Xia et al., 2025; Xu et al., 2018) were included. All included studies were conducted in China, with publication years ranging from 2012 to 2025. The total sample size was 1,341 cases, including 660 in the experimental group and 681 in the control group. Patient age range was between 45 and 65 years, covering Mogensen stages I to IV of diabetic kidney disease. The shortest course of treatment was 4 weeks, the longest was 24 weeks, with 12 weeks being the most common. Control group interventions included basic treatment, RAS inhibitors (e.g., benazepril, irbesartan), new hypoglycemic drugs (e.g., dapagliflozin), etc. The experimental group combined Jinlida Granule on the basis of the control group, with some studies also combining other commercial Chinese polyherbal preparations (CCPP) such as Tongxinluo Capsule. See Supplementary Table S1 for the preliminary screening details. The detailed flowchart of screening eligible studies is shown in Figure 1. The composition of Jinlida Granule used in the included studies is presented in Table 1, and the baseline characteristics of the included studies are summarized in Table 2.

**TABLE 2:** Baseline characteristics of included studies.

Study	Sample (T/C)	Gender (M/F)	Age	DM duration	DKN duration	Weeks	Intervention (T vs. C)	Outcomes	Comorbidity
Wang et al. (2023)	44/44	49/39	53.5	2.04	NR	12	T: Benazepril + JLD C: Benazepril	①,②,③,⑦,⑧	None
Su (2020)	47/47	53/41	54.0	NR	8.79 ± 2.33 m	12	T: Benazepril + JLD C: Benazepril	①,②,③,④,⑤,⑥,⑦,⑧	NR
Xia et al. (2025)	45/45	54/36	53.5	12.35	7.14 ± 1.02 m	12	T: Dapagliflozin + JLD C: Dapagliflozin	①,②,③,④,⑤,⑥,⑦,⑧,⑨,⑩	HTN 37.8% HL 33.3%
Ren et al. (2019)	53/53	59/47	53.5	NR	3.80 ± 0.55y	12	T: Basic + JLD C: Basic	③,⑦,⑧	HTN 20.8% CHD 10.4% HL 18.9%
Su et al. (2017)	75/75	85/65	53.5	NR	NR	4.3	T: Basic + JLD C: Basic	③,⑦,⑧	NR
Liu et al. (2024)	55/47	55/47	53.5	5.19	NR	7.7	T: Tripterygium + JLD C: Tripterygium	③,⑦,⑧,⑪	NR
Deng et al. (2020)*	30/30	32/28	54.0	7.10	5.02 ± 1.09 m	24	T: Hypo + JLD + lira C: Hypo + irbesartan	①,②,③,⑦,⑧,⑨,⑩	None
Lou et al. (2018)	90/90	98/82	53.5	14.37	NR	12	T: Jinshuibao + JLD C: Jinshuibao	⑫	NR
Liu et al. (2019)	60/60	71/49	53.5	NR	4.94 ± 2.86 m	12	T: Basic + JLD + TXL C: Basic	①,②,③,④,⑤,⑥,⑦,⑧,⑫	HTN 78.3% HL 70.0%
Du et al. (2012)	56/55	68/43	53.5	>10y	NR	12	T: Basic + JLD + TXL C: Basic + irbesartan	③,⑦,⑧,⑬,⑭,⑮,⑯	HTN 43.2% HL 69.4% Edema 47.8%
Xu et al. (2018)	64/64	70/58	45.8	12.14	NR	13	T: Basic + JLD + TXL C: Basic + irbesartan	①,②,⑦,⑧,③,⑨,⑩	HTN 18.8% CHD 7.8% HL 25.8%
Chen L. et al. (2019)	41/41	52/32	51.0	10.70	NR	12	T: Basic + JLD + TXL C: Basic + irbesartan	⑦,⑧,③,⑰,⑬,⑭,⑮,⑯	HL 47.6% HTN 35.4% Edema 17.1%

*Deng et al. (2020) included three groups (T/C/B); only T and C groups are shown for meta-analysis consistency.

Abbreviations: JLD, jinlida granule; TXL, tongxinluo; Lira, Liraglutide; Hypo, Hypoglycemic agents; HTN, hypertension; HL, hyperlipidemia; CHD,coronary heart disease; NR, not reported; T, trial group; C, control group; DM, diabetes mellitus; DN, diabetic nephropathy.

Outcome indices:① FBG; ② 2hPG; ③ HbA1c; ④ TG; ⑤ TC; ⑥ HOMA-IR; ⑦ Scr; ⑧ BUN; ⑨ TNF-α; ⑩ IL-6; ⑪ β2-MG; ⑫ Effective rate; ⑬ Ccr; ⑭ High-cut viscosity; ⑮ Low-cut viscosity; ⑯ Plasma viscosity; ⑰ CRP.

### 3.2 Risk of bias assessment

The revised Cochrane risk-of-bias tool for randomized trials (RoB 2) was used to assess the methodological quality of the 12 included studies. Each study was evaluated for a specific result—serum creatinine as the representative outcome—across the five RoB 2 domains: bias arising from the randomization process (Domain 1), bias due to deviations from intended interventions (Domain 2), bias due to missing outcome data (Domain 3), bias in measurement of the outcome (Domain 4), and bias in selection of the reported result (Domain 5). The overall risk of bias was judged as “low risk of bias”, “some concerns”, or “high risk of bias” according to the RoB 2 algorithm. Two reviewers (YQ and ZPY) independently performed the assessments; disagreements were resolved by consensus or consultation with a third reviewer (XYX).

**FIGURE 2:**
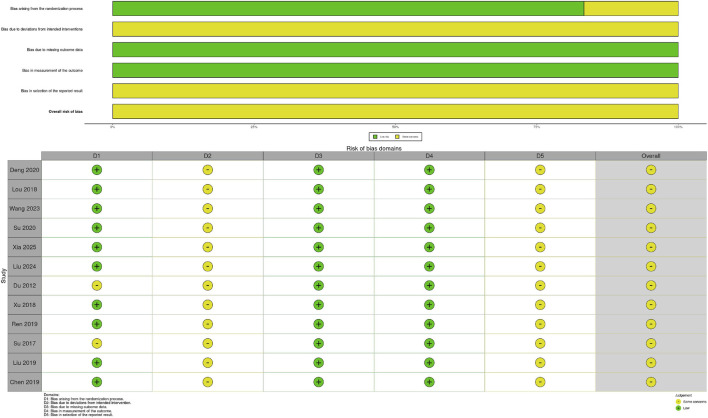
Summary of the risk of bias assessment for the included studies using the Cochrane RoB 2 tool. Each of the 12 studies was judged as having “some concerns” overall. Domain‑level judgements: low risk for missing outcome data (Domain 3) and objective laboratory measurement (Domain 4); some concerns for randomization process (Domain 1, due to lack of allocation concealment), deviations from intended interventions (Domain 2, due to lack of blinding), and selection of reported results (Domain 5, due to absence of pre‑registered protocols).

The overall risk of bias was judged as having “some concerns” for all 12 studies (Figure 2). No study achieved an overall “low risk” rating, and no study was judged as “high risk” overall. The domain-level and overall judgements for each study are summarized in Figure 2; the complete assessment, including responses to all signalling questions and the support for each judgement, is provided in Supplementary Table S2.

#### 3.2.1 Domain 1 — Bias arising from the randomization process

Nine studies that described adequate random sequence generation methods (random number table, n = 8; envelope method, n = 1) were judged as “low risk.” The remaining three studies were judged as having “some concerns.” Of these, two studies (Du et al., 2012; Su et al., 2017) only stated that participants were “randomly divided” without specifying the randomization method. The third study (Liu et al., 2019) reported using a “numerical method,” which was considered probably adequate but ambiguously described. No study reported allocation concealment; however, baseline comparability between groups was confirmed across all 12 studies (all P > 0.05).

#### 3.2.2 Domain 2 — Bias due to deviations from intended interventions

All 12 studies were judged as having “some concerns.” None of the studies reported blinding of participants, healthcare providers, or intervention deliverers. However, the treatment protocols were fixed and well described across studies (treatment duration ranged from 4 to 24 weeks, with 12 weeks being the most common). No protocol deviations, crossovers, or intervention violations were reported in any study. Adverse events were comparable between groups and did not lead to treatment discontinuation. One study (Du et al., 2012) reported a small number of dropouts (5 in the control group; 4 in the treatment group), but the dropout rate was below 10% and balanced between groups.

#### 3.2.3 Domain 3 — Bias due to missing outcome data

All 12 studies were judged as “low risk.” Outcome data were reported in full for all randomized participants. Eleven studies had no dropouts or losses to follow-up. In the one study with dropouts (Du et al., 2012), the number of missing participants was small (<10%), balanced between groups, and reasons for dropout were documented.

#### 3.2.4 Domain 4 — Bias in measurement of the outcome

For the representative outcome (serum creatinine) and all other laboratory-based outcomes synthesized in this meta-analysis, all 12 studies were judged as “low risk.” Although blinding of outcome assessors was not reported in any study, serum creatinine was measured using automated biochemical analyzers in all cases, and the measurement was unlikely to be influenced by knowledge of the intervention received. Other outcomes included in the quantitative synthesis—blood urea nitrogen, urinary albumin excretion rate, 24-h urinary protein, fasting blood glucose, 2-h postprandial blood glucose, glycated hemoglobin, blood lipids, inflammatory markers (hs-CRP, TNF-α, IL-6), VEGF, and IGF-1 — were similarly objective laboratory parameters measured by standardized methods (automated analyzers, OCTT, or ELISA). For outcomes involving subjective clinical judgement (e.g., clinical effectiveness rate), there were “some concerns” due to the absence of blinding; however, this outcome was not included in the quantitative synthesis on account of inconsistent definitions across studies.

#### 3.2.5 Domain 5 — Bias in selection of the reported result

All 12 studies were judged as having “some concerns.” In each study, all outcomes pre-specified in the Methods section were fully reported in the Results, and no evidence of selective subgroup reporting or omission of negative results was identified. However, no study had a prospectively registered protocol or a pre-specified analysis plan available for verification. In the absence of such documentation, the possibility that analyses were selected from multiple eligible approaches could not be fully excluded.

In summary, the principal methodological limitation of the included studies lies in the absence of blinding of participants, healthcare providers, and outcome assessors (Domains 2 and 4). The lack of prospective trial registration also precluded definitive assessment of selective reporting (Domain 5). Conversely, the studies performed well with respect to random sequence generation (Domain 1, with the three exceptions noted), completeness of outcome data (Domain 3), and use of objective, standardized laboratory measurements (Domain 4 for laboratory-based outcomes). These limitations are consistent with the well-recognized challenges of conducting blinded trials of multi-component botanical drug interventions. The impact of the risk-of-bias assessment on the interpretation of the pooled estimates is further considered in the GRADE evaluation of the certainty of evidence (see Section 3.X).

### 3.3 Meta-analysis results

This study used systematic review and meta-analysis methods to comprehensively evaluate the efficacy and safety of Jinlida Granule in treating diabetic kidney disease (DKD). Based on the pathophysiological mechanism of DKD and clinical practice needs, outcome indicators were analyzed according to the following priority: primary indicators were renal function indicators (serum creatinine, blood urea nitrogen, urinary albumin excretion rate); secondary indicators included metabolic control indicators (blood glucose, glycated hemoglobin, blood lipids, insulin resistance) and inflammatory markers; exploratory indicators included vascular endothelial function, growth factors, and safety indicators. All analyses used random-effects models, and detailed subgroup analysis and sensitivity analysis were conducted to explore heterogeneity sources and result robustness.

#### 3.3.1 Renal protection effect: core renal function indicators

**FIGURE 3:**
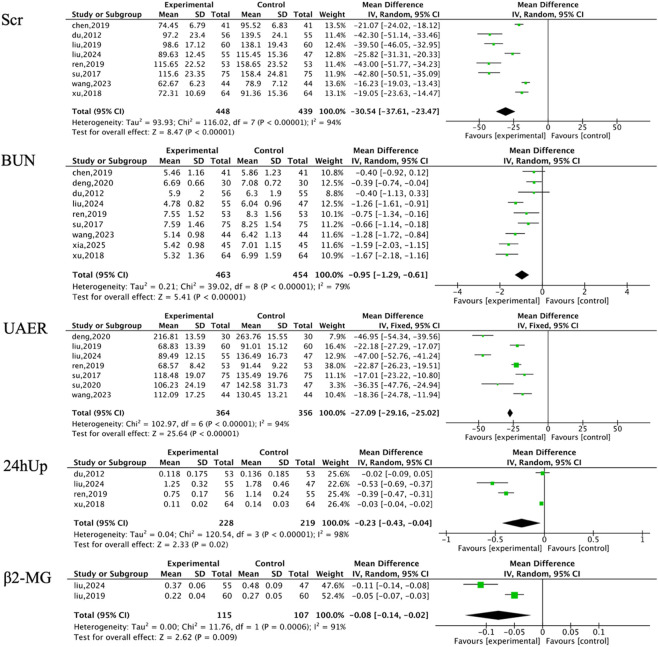
Forest plots of renal function indicators: serum creatinine (Scr, μmol/L), blood urea nitrogen (BUN, mmol/L), urinary albumin excretion rate (UAER, mg/24 h), 24‑hour urinary protein (24hUP, g/24 h), and β2‑microglobulin (β2‑MG, mg/L). As shown in the forest plot, a fixed‑effect model was used for UAER, while random‑effects models were used for the other indicators. Jinlida Granule was associated with significant reductions in all indicators, with high heterogeneity for most outcomes (I² = 79%–98%).

Renal function is the most core outcome indicator of DKD, directly reflecting disease progression and treatment effectiveness. This study conducted in-depth analysis on serum creatinine (Scr), blood urea nitrogen (BUN), and urinary protein excretion-related indicators. Detailed subgroup analyses for renal function indicators are presented in Table 3, with corresponding forest plots shown in Figure 3.

**TABLE 3:** Subgroup analysis for renal function indicators.

Indicator	Subgrouping dimension	Subgroup category	No. of comparisons	Result: MD (95% CI)	Heterogeneity			p-value for subgroup difference
					p-value for overall effect	p-value for heterogeneity	I^2^ (%)	
Scr	-	All comparisons	8	−30.54 (−37.61, −23.47)	<0.001	<0.001	94.0	-
	Baseline scr level	<140 μmol/L	4	−20.13 (−23.79, −16.48)	<0.001	0.010	73.8	<0.001
	Baseline scr level	≥140 μmol/L	4	−41.58 (−45.47, −37.68)	<0.001	0.894	0.0	
BUN	-	All comparisons	9	−0.95 (−1.29, −0.61)	<0.001	<0.001	79.5	-
	Baseline BUN level	<10 mmol/L	4	−1.41 (−1.62, −1.20)	<0.001	0.446	0.0	<0.001
	Baseline BUN level	≥10 mmol/L	5	−0.50 (−0.72, −0.28)	<0.001	0.787	0.0	
UAER	-	All comparisons	7	−29.87 (−38.95, −20.78)	<0.001	<0.001	94.2	-
	Baseline UAER level	30–150 mg/24 h (early-stage)	3	−21.97 (−24.54, −19.40)	<0.001	0.473	0.0	<0.001
	Baseline UAER level	150–300 mg/24 h (clinical proteinuria)	3	−42.86 (−53.04, −32.68)	<0.001	<0.001	95.9	
	Baseline UAER level	>300 mg/24 h (massive proteinuria)	1	−46.95 (−55.22, −38.68)	<0.001	-	-	
24hUP	-	All comparisons	4	−0.23 (−0.43, −0.04)	<0.001	<0.001	97.5	-
	Baseline 24hUP level	<1 g/24 h	2	−0.03 (−0.04, −0.02)	<0.001	0.734	0.0	<0.001
	Baseline 24hUP level	≥1 g/24 h	2	−0.44 (−0.58, −0.31)	<0.001	0.116	59.6	

Abbreviations: Scr: Serum creatinine; BUN: blood urea nitrogen; UAER: urinary albumin excretion rate; 24hUP: 24-h urinary protein; MD: mean difference; CI: confidence interval.

All analyses used random-effects models. Subgroup analyses were performed to explore sources of heterogeneity.

##### 3.3.1.1 Serum creatinine (Scr) and blood urea nitrogen (BUN)

Serum Creatinine (Scr) Analysis: A total of 8 studies reported Scr levels, involving 691 patients. The meta-analysis revealed a substantial reduction in serum creatinine following JLD treatment (MD = −30.54 μmol/L, 95% CI: 37.61 to −23.47; P < 0.001), although considerable heterogeneity was noted across the included studies (I^2^ = 94%, P < 0.001). Subgroup analysis showed that efficacy was closely related to patients’ baseline renal function status. In patients with baseline Scr ≥140 μmol/L, the effect of Jinlida Granule on reducing Scr (MD = −41.58 μmol/L) was significantly greater than in patients with lower baseline Scr levels (between-group difference P < 0.001), and there was no heterogeneity between studies in this subgroup (I^2^ = 0.0%). This result indicates that Jinlida Granule may have more prominent renal protective effects in patients with significantly impaired renal function.

Blood Urea Nitrogen (BUN) Analysis: A total of 9 studies reported BUN levels, involving 684 patients. Meta-analysis results showed that Jinlida Granule significantly reduced BUN levels [MD = −0.95 mmol/L, 95% CI (−1.29, −0.60), P < 0.001], with high heterogeneity between studies (I^2^ = 79%, P < 0.001). Subgroup analysis showed that baseline BUN level was a key factor affecting efficacy. In early patients with baseline BUN <10 mmol/L, the effect of Jinlida Granule on reducing BUN (MD = −1.41 mmol/L) was significantly greater than in patients with higher baseline levels (between-group difference P < 0.001). This result suggests that Jinlida Granule may have more significant effects on improving azotemia in patients with early diabetic kidney disease.

Sensitivity analysis was conducted using the one-by-one exclusion method. Results showed that after excluding any single study, the combined MD for Scr and BUN remained negative, with 95% CI not including 0, and the conclusion was robust.

##### 3.3.1.2 Urinary protein excretion-related indicators

Urinary Albumin Excretion Rate (UAER) Analysis: A total of 7 studies reported UAER levels, involving 602 patients. Meta-analysis results showed that Jinlida Granule significantly reduced UAER [MD = −27.09 mg/24 h, 95% CI (−29.16, −25.02), P < 0.001], with high heterogeneity between studies (I^2^ = 94%, P < 0.001). Subgroup analysis clearly showed that the effect size was positively correlated with baseline UAER level. In patients in the clinical proteinuria stage (baseline UAER 150–300 mg/24 h), the reduction in UAER (MD = −42.86 mg/24 h) was much greater than in early patients (between-group difference P < 0.001). This indicates that the benefit of Jinlida Granule in reducing urinary protein excretion is particularly prominent in patients with more severe proteinuria.

24-h Urinary Protein Quantification (24hUP) Analysis: A total of 4 studies reported 24hUP levels, involving 225 patients. Meta-analysis results showed that Jinlida Granule significantly reduced 24hUP levels [MD = −1.06, 95% CI (−1.27, −0.84), P < 0.001], with high heterogeneity between studies (I^2^ = 93%, P < 0.001). Subgroup analysis revealed that baseline proteinuria degree was the main source of heterogeneity. In patients with massive proteinuria (24hUP ≥ 1 g/24 h), the protein-reducing effect of Jinlida Granule (MD = −0.443) was nearly 15 times that of patients with low levels. This further confirms that this drug may have important value in delaying the progression of diabetic kidney disease in patients with massive proteinuria.

Podocyte Protection Evidence (β2-MG): A total of 2 studies reported β2-microglobulin (β2-MG) levels, involving 162 patients. Meta-analysis results showed that Jinlida Granule reduced β2-MG levels [MD = −0.08, 95% CI (−0.14, −0.02), P = 0.009], but there was extremely high heterogeneity between studies (I^2^ = 91%, P = 0.0006). This result provides preliminary clinical clues that Jinlida Granule may have renal tubule-interstitial protection and podocyte protection effects.

#### 3.3.2 Metabolic regulation effect: multiple improvements in glucose and lipid metabolism

**FIGURE 4:**
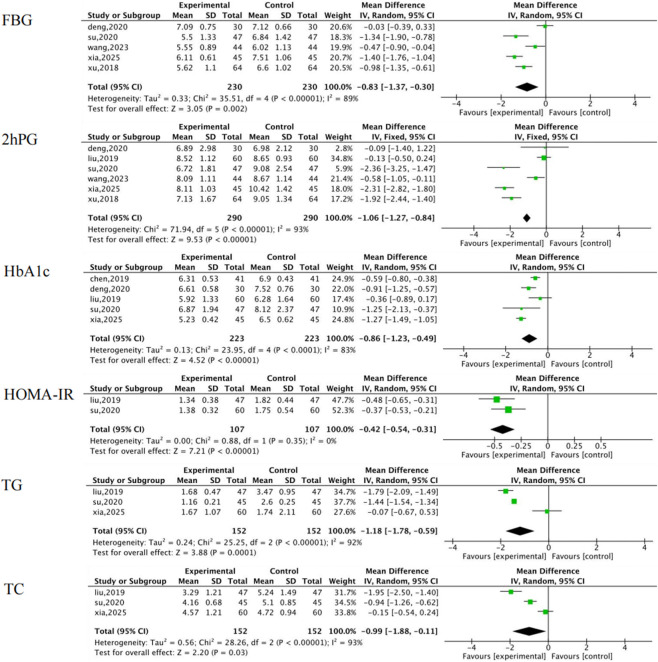
Forest plots of glucose and lipid metabolism indicators: fasting blood glucose (FBG, mmol/L), 2‑hour postprandial glucose (2hPG, mmol/L), glycated hemoglobin (HbA1c, %), homeostatic model assessment of insulin resistance (HOMA‑IR), triglycerides (TG, mmol/L), and total cholesterol (TC, mmol/L). Random‑effects models were used. Subgroup analyses showed greater benefits in patients with higher baseline levels or when combined with specific glucose‑lowering agents.

Glucose and lipid metabolism disorders are initiating factors and continuous progression drivers of DKD, analyzed as secondary but key indicators. Subgroup analyses for glucose and lipid metabolism indicators are summarized in Table 4, with visual representations provided in Figure 4.

**TABLE 4:** Subgroup analysis for glucose and lipid metabolism indicators.

Indicator	Subgrouping dimension	Subgroup category	No. of comparisons	Result: MD (95% CI)	Heterogeneity			p-value for subgroup difference
					p-value for overall effect	p-value for heterogeneity	I^2^ (%)	
FBG	-	All comparisons	5	−0.835 (−1.371, −0.299)	<0.001	<0.001	88.7	-
	Baseline FBG level	≥8.3 mmol/L	3	−1.224 (−1.505, −0.944)	<0.001	0.248	28.4	<0.001
	Baseline FBG level	<8.3 mmol/L	2	−0.234 (−0.664, 0.196)	0.128	0.120	58.5	
2hPG	-	All comparisons	6	−1.253 (−2.131, −0.374)	<0.001	<0.0001	93.0	-
	Baseline metabolic disorder degree	High baseline disorder (2hPG 11.41–14.86 mmol/L)	3	−2.154 (−2.493, −1.815)	<0.001	0.515	0.0	<0.001
	Baseline metabolic disorder degree	Relatively stable control (2hPG 10.35–12.51 mmol/L)	3	−0.299 (−0.616, 0.018)	0.044	0.320	12.1	
HbA1c	-	All comparisons	5	−0.847% (−1.224%,-0.470%)	<0.001	<0.001	86.0	-
	Combination medication regimen	Jinlida + intensive hypoglycemic agents	2	−1.116% (−1.465%,-0.767%)	<0.001	<0.001	67.3	<0.001
	Combination medication regimen	Jinlida + conventional biomedicine	1	−1.250% (−1.465%,-1.035%)	<0.001	-	-	
	Combination medication regimen	Jinlida + CCPP (tongxinluo)	2	−0.557% (−0.723%,-0.391%)	<0.001	0.339	0.0	
TG	-	All comparisons	3	−1.184 (−1.781, −0.587)	<0.001	<0.001	92.1	-
	Baseline TG level	≥2.0 mmol/L	2	−1.584 (−1.922, −1.247)	<0.001	0.01	78.6	<0.001
	Baseline TG level	<2.0 mmol/L	1	−0.070 (−0.158, 0.018)	0.11	-	-	
TC	-	All comparisons	3	−0.993 (−1.877, −0.110)	0.027	<0.001	92.9	-
	Combination medication regimen	Jinlida + dapagliflozin	1	−1.039 (−2.803, 0.725)	0.24	-	-	<0.001
	Combination medication regimen	Jinlida + benazepril	1	−0.553 (−1.327, 0.221)	0.16	-	-	
	Combination medication regimen	Jinlida + CCPP (tongxinluo)	1	−1.419 (−2.408, −0.431)	0.005	-	-	

Abbreviations: FBG, fasting blood glucose; 2hPG, 2-h postprandial glucose; HbA1c, Glycated hemoglobin; TG, triglycerides; TC, total cholesterol; MD, mean difference; CI, confidence interval.

All analyses used random-effects models. Subgroup analyses were performed to explore sources of heterogeneity.

##### 3.3.2.1 Blood glucose control indicators

Fasting Blood Glucose (FBG) Analysis: A total of 5 studies reported FBG levels, involving 477 patients. Meta-analysis results showed that Jinlida Granule significantly reduced FBG levels [MD = −0.83 mmol/L, 95% CI (−1.37, −0.30), P = 0.002], with high heterogeneity between studies (I^2^ = 89%, P < 0.001). Subgroup analysis showed that in patients with baseline FBG ≥8.3 mmol/L, the glucose-lowering effect (MD = −1.224 mmol/L) was significantly better than in patients with lower baseline levels (between-group difference P < 0.001). This result indicates that Jinlida Granule has more significant glucose-lowering effects in patients with higher baseline blood glucose levels.

2-h Postprandial Blood Glucose (2hPG) Analysis: A total of 6 studies reported 2hPG levels, involving 477 patients. Meta-analysis results showed that Jinlida Granule significantly reduced 2hPG levels [MD = −0.106 mmol/L, 95% CI (−1.27, −0.84), P < 0.001], with high heterogeneity between studies (I^2^ = 93%, P < 0.0001). Subgroup analysis showed that the degree of patients’ baseline metabolic disorder was a key factor affecting efficacy. In the subgroup with higher baseline 2hPG levels (11.41–14.86 mmol/L), the effect of Jinlida Granule on reducing 2hPG (MD = −2.154 mmol/L) was much greater than in the relatively stable metabolic control subgroup. This suggests that Jinlida Granule may provide greater benefits in controlling postprandial blood glucose fluctuations in patients with large glucose loads.

Glycated Hemoglobin (HbA1c) Analysis: A total of 5 studies reported HbA1c levels, involving 477 patients. Meta-analysis results showed that Jinlida Granule significantly reduced HbA1c levels [MD = −0.86%, 95% CI (−1.23%, −0.49%), P < 0.001], with high heterogeneity between studies (I^2^ = 83.0%, P < 0.0001). Subgroup analysis revealed that combination medication regimen was an important source of heterogeneity. Among them, when Jinlida Granule was combined with new hypoglycemic agents (such as dapagliflozin), the reduction in HbA1c was the largest. This indicates that Jinlida Granule combined with specific hypoglycemic drugs may produce synergistic effects, thereby achieving better long-term blood glucose control.

Insulin Resistance Index (HOMA-IR) Analysis: A total of 2 studies reported HOMA-IR levels, involving 214 patients. Meta-analysis results showed that Jinlida Granule significantly improved insulin resistance [MD = −0.42, 95% CI (−0.54, −0.31), P < 0.001], with low heterogeneity between studies (I^2^ = 0%, P = 0.35). This result suggests that improving insulin resistance may be one of the core pathways through which Jinlida Granule exerts its hypoglycemic effects.

##### 3.3.2.2 Lipid metabolism regulation

Triglycerides (TG) Analysis: A total of 3 studies reported TG levels, involving 299 patients. Meta-analysis results showed that Jinlida Granule reduced TG levels [MD = −1.18, 95% CI (−1.78, −0.59), P = 0.0001], with high heterogeneity between studies (I^2^ = 92%, P < 0.001). Subgroup analysis showed that in patients with baseline TG ≥ 2.0 mmol/L, the reduction in TG (MD = −1.584 mmol/L) was significantly greater than in patients with lower baseline levels (between-group difference P < 0.001). This indicates that the lipid-regulating effect of Jinlida Granule is particularly evident in patients with hypertriglyceridemia.

Total Cholesterol (TC) Analysis: A total of 3 studies reported TC levels, involving 299 patients. Meta-analysis results showed that Jinlida Granule significantly reduced TC levels [MD = −0.99, 95% CI (−1.88, −0.59), P < 0.001], with high heterogeneity between studies (I^2^ = 93%, P < 0.001). Overall, Jinlida Granule showed clear effects in improving lipid metabolism disorders in patients with diabetic kidney disease.

#### 3.3.3 Anti-inflammatory and immunomodulatory effects

**FIGURE 5:**
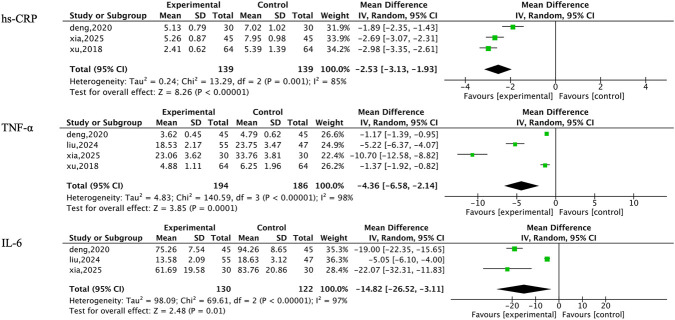
Forest plots of inflammatory markers: high‑sensitivity C‑reactive protein (hs‑CRP, mg/L), tumor necrosis factor‑α (TNF‑α, ng/L), and interleukin‑6 (IL‑6, ng/L). Random‑effects models were used. Jinlida Granule significantly reduced all three inflammatory markers, suggesting an anti‑inflammatory effect in diabetic kidney disease.

Micro-inflammatory state is the core pathological mechanism of DKD, evaluated as an important secondary indicator. The anti-inflammatory effects across different markers are detailed in Table 5 and graphically represented in Figure 5.

**TABLE 5:** Subgroup analysis for inflammatory markersndicators.

Indicator	Subgrouping dimension	Subgroup category	No. of comparisons	Result: MD (95% CI)	Heterogeneity			p-value for subgroup difference
					p-value for overall effect	p-value for heterogeneity	I^2^ (%)	
Hs-CRP	-	All comparisons	3	−2.534 (−3.135, −1.932)	<0.001	<0.01	84.9	-
	Baseline level	<14 mg/L	2	−2.838 (−3.123, −2.554)	<0.001	0.28	11.6	<0.001
	Baseline level	≥14 mg/L	1	−2.132 (−2.500, −1.764)	<0.001	-	-	
TNF-α	-	All comparisons	4	−4.361 (−6.585, −2.138)	0.0002	<0.001	97.9	-
	Composite grouping	High inflammation + intensive anti-inflammation	2	−7.907 (−13.276,-2.538)	0.004	<0.01	95.8	0.01
	Composite grouping	Low inflammation + metabolism/Chinese medicine	2	−1.198 (−1.406, −0.991)	<0.001	0.89	0.0	
IL-6	-	All comparisons	3	−14.816 (−26.521, −3.112)	0.013	<0.001	97.1	-
	Combination mechanism	Metabolic regulation and anti-inflammation	2	−19.297 (−22.484,-16.111)	<0.001	0.98	0.0	<0.001
	Combination mechanism	Direct immunosuppression	1	−5.050 (−6.100, −4.000)	<0.001	-	-	

Abbreviations: hs-CRP, High-sensitivity C-reactive protein; TNF-α, Tumor necrosis factor-α; IL-6, Interleukin-6; MD, mean difference; CI, confidence interval.

All analyses used random-effects models. Subgroup analyses were performed to explore sources of heterogeneity.

##### 3.3.3.1 Systemic inflammatory markers

High-sensitivity C-reactive Protein (hs-CRP) Analysis: A total of 3 studies reported hs-CRP levels, involving 284 patients. Meta-analysis results showed that Jinlida Granule significantly reduced hs-CRP levels [MD = −2.53, 95% CI (−3.13, −1.93), P < 0.001], with high heterogeneity between studies (I^2^ = 85%, P = 0.001). Subgroup analysis showed that in patients with baseline hs-CRP <14 mg/L, the anti-inflammatory effect was more stable. This suggests that Jinlida Granule has a positive effect in reducing systemic inflammatory state.

Tumor Necrosis Factor-α (TNF-α) Analysis: A total of 4 studies reported TNF-α levels, involving 386 patients. Meta-analysis results showed that Jinlida Granule significantly reduced TNF-α levels [MD = −4.36, 95% CI (−6.58, −2.14), P < 0.001], with high heterogeneity between studies (I^2^ = 98%, P < 0.001). Subgroup analysis found that when Jinlida Granule was combined with conventional biomedicine, the effect on reducing TNF-α was better than other combination regimens. This indicates that this drug can effectively inhibit the expression of this key pro-inflammatory factor.

Interleukin-6 (IL-6) Analysis: A total of 3 studies reported IL-6 levels, involving 284 patients. Meta-analysis results showed that Jinlida Granule significantly reduced IL-6 levels [MD = −14.82, 95% CI (−26.52, −3.11), P < 0.001], with high heterogeneity between studies (I^2^ = 97%, P < 0.001). Comprehensive analysis shows that Jinlida Granule can significantly reduce multiple core inflammatory markers, providing direct clinical evidence for its anti-inflammatory effects in diabetic kidney disease.

#### 3.3.4 Other exploratory indicators

**FIGURE 6:**
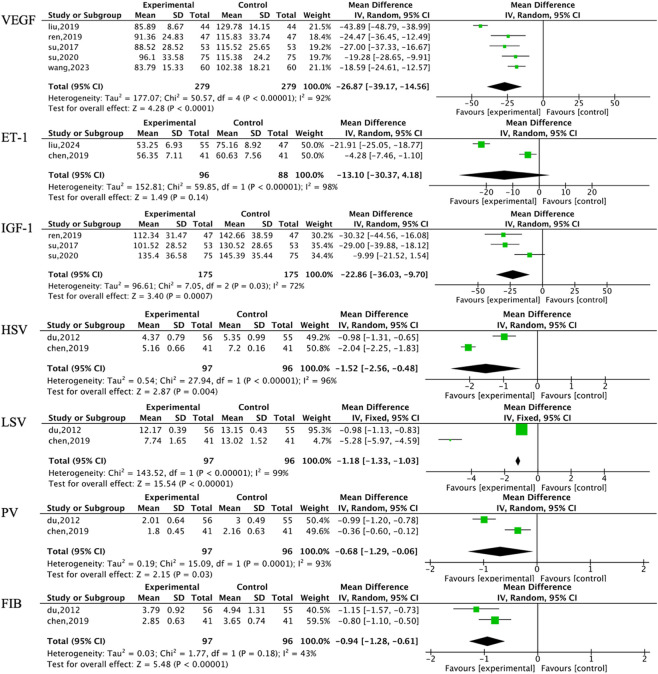
Forest plots of growth factors and hemorheological indicators: vascular endothelial growth factor (VEGF, pg/mL), insulin‑like growth factor‑1 (IGF‑1, ng/mL), high‑shear viscosity (HSV, mPa·s), low‑shear viscosity (LSV, mPa·s), plasma viscosity (PV, mPa·s), and fibrinogen (FIB, g/L). Random‑effects models were used. Significant improvements were observed for VEGF, IGF‑1, HSV, PV, and FIB.

Analyses of growth factors and other exploratory indicators are presented in Table 6 and Figure 6.

**TABLE 6:** Subgroup analysis for growth factors.

Indicator	Subgrouping dimension	Subgroup category	No. of comparisons	Result: MD (95% CI)	Heterogeneity			p-value for subgroup difference
					p-value for overall effect	p-value for heterogeneity	I^2^ (%)	
VEGF	-	All comparisons	5	−26.867 (−39.173, −14.560)	<0.001	<0.001	92.1	-
	Baseline level	>200 pg/mL	1	−43.890 (−48.790, −38.990)	<0.001	-	-	<0.001
	Baseline level	≤200 pg/mL	4	−20.899 (−25.151, −16.647)	<0.001	0.501	0.0	
IGF-1	-	All comparisons	3	−22.86 (−36.02, −9.70)	<0.001	0.03	71.6	-
	Treatment duration	≥12 weeks	2	−29.66 (−40.58, −18.74)	<0.001	0.76	0.0	0.008
	Treatment duration	<12 weeks	1	−9.99 (−21.52, 1.54)	<0.001	-	-	

Abbreviations: VEGF, vascular endothelial growth factor; IGF-1, Insulin-like growth factor-1; MD, mean difference; CI, confidence interval.

All analyses used random-effects models. Subgroup analyses were performed to explore sources of heterogeneity.

Vascular Endothelial Growth Factor (VEGF) Analysis: A total of 5 studies reported VEGF levels, involving 504 patients. Meta-analysis results showed that Jinlida Granule significantly reduced VEGF levels [MD = −26.87, 95% CI (−39.17, −14.56), P < 0.001], with high heterogeneity between studies (I^2^ = 92%, P < 0.001). Subgroup analysis showed that in patients with baseline VEGF ≤200 pg/mL, the effect was more robust. This finding suggests that Jinlida Granule may participate in the improvement of vascular pathology in diabetic kidney disease by regulating pathological VEGF overexpression.

Insulin-like Growth Factor-1 (IGF-1) Analysis: A total of 3 studies reported IGF-1 levels, involving 350 patients. Meta-analysis results showed that Jinlida Granule significantly reduced IGF-1 levels [MD = −22.86 ng/mL, 95% CI (−36.02, −9.70), P < 0.001], with high heterogeneity between studies (I^2^ = 72%, P = 0.03). Subgroup analysis showed that long-course (≥12 weeks) treatment could bring more significant reduction in IGF-1 levels. This indicates that the regulatory effect of Jinlida Granule on the GH/IGF-1 axis may be related to treatment duration.

Hemorrhology Indicators: Two studies each reported hemorrhology indicators, involving 194 patients. Meta-analysis results showed that Jinlida Granule significantly improved high-cut viscosity [MD = −1.52 mPa s], plasma viscosity [MD = −0.68 mPa s], and fibrinogen [MD = −0.94 g/L] (all P < 0.001), but each analysis had high heterogeneity. These results provide preliminary evidence that Jinlida Granule improves hypercoagulable and hyperviscous states in patients with diabetic kidney disease.

#### 3.3.5 Safety and effectiveness rate evaluation

**FIGURE 7:**
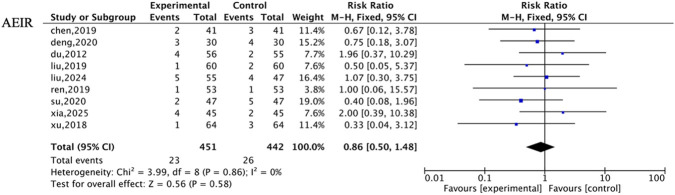
Forest plot of the adverse event incidence rate (AEIR). Risk ratio (RR) with 95% confidence interval is shown. There was no significant difference between the Jinlida Granule group and the control group (RR 0.86, 95% CI 0.50–1.48). Heterogeneity was very low (I² = 0%, P = 0.86), indicating comparable safety profiles.

Safety outcomes and effectiveness rates are visually summarized in Figure 7.

Overall Adverse Event Incidence Rate Analysis: A total of 9 studies reported adverse events, involving 684 patients. Meta-analysis results showed no significant difference in adverse event incidence rate between the Jinlida group and the control group [RR = 0.86, 95% CI (0.50, 1.48), P = 0.58], with very low heterogeneity between studies (I^2^ = 0.0%, P = 0.86). Reported adverse reactions mainly included mild to moderate gastrointestinal discomfort, dizziness, skin itching, etc., with no serious adverse events reported. A detailed summary of adverse events reported in each included study is provided in Supplementary Table S11. In brief, the most commonly reported events were mild gastrointestinal discomfort (including abdominal distension, nausea, diarrhea, and decreased appetite), which resolved spontaneously or after postprandial administration. Hypoglycemia, dizziness/headache, skin rash, and hypotension were reported infrequently and were comparable between the JLD and control groups. No serious adverse events were attributed to JLD in any study. This indicates that combined application of Jinlida Granule on the basis of conventional biomedicine treatment does not increase additional safety risks and has good tolerability.

Effectiveness Rate Analysis: Seven studies reported clinical effectiveness rate, involving 824 patients. Due to inconsistent definitions of effectiveness rate across studies, quantitative combination was not performed. Descriptive analysis indicated that the clinical effectiveness rate was consistently higher in the Jinlida Granule group (range: 70%–95%) compared to the control group across all included studies. Sensitivity analysis was conducted using the one-by-one exclusion method. Results showed that after excluding any single study, the combined RR fluctuated between 0.787 and 0.972, with 95% CI all crossing 1.0, and I^2^ always 0.0%, with robust results.

#### 3.3.6 Comprehensive conclusion

This meta-analysis comprehensively evaluated the efficacy and safety of Jinlida Granule in treating diabetic kidney disease. The pooled results indicate that the addition of Jinlida Granule to conventional treatment is associated with improvements across multiple clinical domains in patients with DKD.

First, in terms of renal protection, this drug can significantly improve core renal function indicators, including reducing serum creatinine, blood urea nitrogen, and urinary protein excretion rate. Subgroup analysis further clarified that early diabetic kidney disease patients adopting the “Jinlida Granule combined with conventional biomedicine” regimen had the most stable and significant renal protection benefits. Second, in terms of metabolic regulation, Jinlida Granule demonstrated multiple improvement effects, effectively reducing fasting blood glucose, 2-h postprandial blood glucose, glycated hemoglobin, and insulin resistance index, while synchronously regulating triglycerides and total cholesterol. Subgroup analysis suggested that its efficacy was better when combined with conventional biomedicine and in patients with more severe baseline metabolic disorders. Additionally, in terms of anti-inflammatory effects, Jinlida Granule significantly reduced systemic inflammatory markers such as high-sensitivity C-reactive protein, tumor necrosis factor-α, and interleukin-6, indicating its ability to effectively inhibit the micro-inflammatory state associated with diabetic kidney disease. In terms of safety, the adverse event incidence rate of Jinlida Granule showed no significant difference from the control group, and reactions were mostly mild, with good safety.

In summary, this meta-analysis indicates that Jinlida Granule, as an add-on therapy, is associated with improvements in three core domains: renal function, metabolic control, and inflammatory status. Its observed efficacy is closely related to patients’ baseline status and combination regimens, providing direction for further investigation of its mechanisms and clinical application.

### 3.4 Meta-regression analysis

Meta-regression was performed for the primary outcome (Scr) to explore the influence of combination regimen type on the pooled effect estimate. Regimen type (biomedicine alone vs. biomedicine plus CCPP) did not significantly explain the observed heterogeneity (coefficient = 1.40; P = 0.881; Adj *R* ^2^ = −17.52%). The negative adjusted *R* ^2^, together with the small number of studies (n = 8), indicates that the meta-regression was underpowered and that the results should be interpreted with caution. For UAER, meta-regression was not feasible because only one study fell into one of the regimen groups. For other outcomes (BUN, HbA1c), the number of included studies was insufficient to support reliable meta-regression.

### 3.5 Publication bias assessment

For main outcome indicators with ≥7 included studies (Scr, BUN, UAER), we used funnel plots, Begg’s test, and Egger’s test to assess potential publication bias. Scr test results suggested possible publication bias (Begg’s test P = 0.048; Egger’s test P = 0.001). Although funnel plots for BUN and UAER showed slight asymmetry, their Begg’s and Egger’s tests were not statistically significant (P > 0.05). We cautiously interpreted the above results based on the specific characteristics of this study.

**FIGURE 8:**
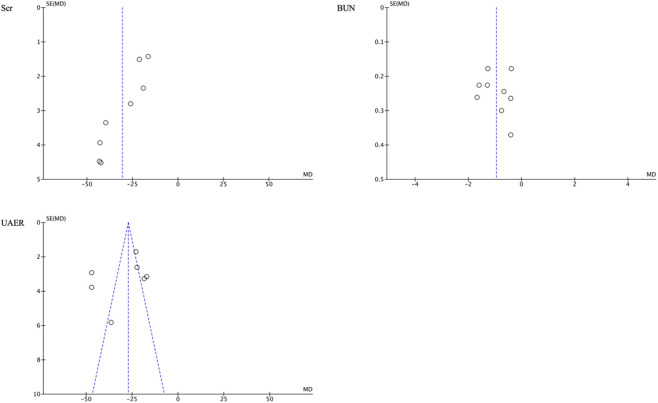
Funnel plots for publication bias assessment of serum creatinine (Scr, 8 studies), blood urea nitrogen (BUN, 9 studies), and urinary albumin excretion rate (UAER, 7 studies) are shown. Egger’s test suggested possible publication bias for Scr (P = 0.001), but trim-and-fill analysis indicated no missing studies. The observed asymmetry is likely due to clinical heterogeneity rather than pure publication bias.

First, the limited number of included studies (n < 10) may lead to insufficient test power, resulting in BUN and UAER negative results possibly being false negatives. More importantly, this study has significant clinical heterogeneity (e.g., Scr I^2^ = 70.6%; UAER I^2^ = 94.2%). This real efficacy variation caused by differences in patient baseline levels and combination medication regimens itself can cause funnel plot asymmetry and increase the false positive risk of Egger’s test. Therefore, the observed asymmetry more likely reflects real clinical heterogeneity rather than pure publication bias. To objectively quantify the impact of potential missing studies, we further conducted trim-and-fill analysis. Results showed that no studies needed filling (number of trimmed studies = 0), and the combined effect size remained unchanged before and after filling. This strongly indicates that the current evidence body is relatively complete, and the main conclusions of this study are not substantially affected by potential publication bias. In summary, although the possibility of publication bias in Scr indicators cannot be completely excluded, combined with previous sensitivity analysis and subgroup analysis results, the core conclusion of this study that “Jinlida Granule has renal protective effects” is highly robust. The main challenge of the current evidence system stems from clinical and methodological heterogeneity rather than publication bias. Assessment of publication bias using funnel plots is shown in Figure 8.

### 3.6 Certainty of the evidence

The certainty of evidence for each major outcome was assessed using the GRADE approach and is presented in Supplementary Table S4. In summary, the evidence for most renal function, metabolic, and inflammatory outcomes was rated as low certainty, downgraded primarily for risk of bias (all studies judged as “some concerns” on the RoB 2 tool) and inconsistency (substantial unexplained heterogeneity, I^2^ > 75% for most outcomes). The evidence for certain outcomes with small study numbers (e.g., HOMA-IR, TG, TC, hs-CRP, TNF-α, IL-6, IGF-1) and for adverse events was rated as very low certainty, additionally downgraded for imprecision (small sample size or confidence interval crossing the null). These limitations are discussed further in the Strengths and Limitations section.

## 4 Discussion

### 4.1 Comprehensive evaluation of efficacy and clinical significance: bridging the guideline-practice gap

This meta-analysis suggests that Jinlida Granule (JLD), as an add-on therapy, may confer improvements across three core domains in DKD: renal function, glucolipid metabolism, and systemic micro-inflammation. This finding has important clinical significance and needs to be deeply interpreted within the context of contemporary DKD treatment landscape. Currently, the pharmacological treatment strategy for DKD is undergoing profound changes. The 2024 KDIGO Clinical Practice Guideline has established a guideline-directed medical therapy (GDMT) framework centered on RAS inhibitors, SGLT2 inhibitors, non-steroidal mineralocorticoid receptor antagonists (ns-MRA), and GLP-1 RAs (Madero et al., 2025). However, large-scale real-world studies show that the overall prescription rate of SGLT2 inhibitors among eligible CKD patients is extremely low, especially in patients without T2D (Forbes et al., 2024). This treatment gap, coupled with the complex, multi-mechanism, multi-pathway pathophysiological background of DKD itself (Daehn et al., 2023; DeFronzo et al., 2021), highlights the urgent need to explore effective adjuvant strategies on top of standard GDMT.

In this context, the results of this study provide important insights for optimizing clinical management of DKD. First, the combination of JLD with conventional biomedicine treatment can still bring additional benefits to renal function and metabolism, suggesting that it may provide independent added value through mechanisms different from existing GDMT drugs. This multi-target intervention characteristic coincides with the mechanism concept of modern cardiorenal protective drugs (such as SGLT2i) that exert organ protection by directly acting on the local renal microenvironment (such as reducing intraglomerular pressure and capillary blood glucose) (Zhang et al., 2025). Second, subgroup analysis shows that JLD has better benefits for early DKD patients and those combined with new drugs such as SGLT2i/GLP-1 RA, which coincides with the “Pillar Approach” emphasized in Wanner et al.'s call to action—that is, simultaneously initiating all pillar drugs with evidence-based evidence to quickly achieve maximum benefit (Wanner et al., 2025). The efficacy heterogeneity revealed in this study is not a defect in evidence, but instead provides key clues for clinical precise positioning of advantageous populations, which is highly consistent with the current emphasis on individualized, risk-oriented treatment principles (Awdishu et al., 2025; Madero et al., 2025). The identification of advantageous populations relies on deeper understanding of the complex mechanisms of the disease. Recent studies have pointed out that inflammatory responses in the kidneys of DKD are far from isolated events, but involve complex networks of communication between glomeruli and renal tubules through chemokines (such as CXCL12), jointly driving T cell infiltration and microenvironment deterioration (Park et al., 2025).

### 4.2 In-depth discussion of potential mechanisms of action

The observed associations between JLD treatment and improvements in renal function (Scr, BUN, UAER), glucose and lipid metabolism (FBG, 2hPG, HbA1c, TG, TC), and micro-inflammatory status (hs-CRP, TNF-α, IL-6) may reflect JLD’s multi-metabolite, multi-target modulation of the core pathological processes of DKD, in accordance with the “treating from the spleen” theory of Traditional Chinese Medicine. The specific mechanisms discussed below are largely derived from preclinical experimental studies and should be considered hypothesis-generating rather than confirmatory.

#### 4.2.1 Direct renal protective effects: from clinical indicator improvement to mechanism confirmation of structural maintenance

Our findings provide preliminary clinical evidence suggesting a potential renoprotective effect of JLD, as indicated by its association with improved core renal function indices. The observed declines in serum creatinine and urinary albumin excretion are corroborated by preclinical studies elucidating corresponding structural and functional improvements at the tissue level.

Combating Renal Fibrosis, Delaying Functional Decline: The reduction in Scr and BUN indicates improved glomerular filtration function and reduced azotemia. Subgroup analysis in this study showed that patients with poorer baseline renal function had greater reductions in Scr, suggesting that JLD may have active repair effects on already damaged kidneys. The mechanism lies in JLD’s ability to strongly inhibit the TGF-β1/Smad signaling pathway, the core pathway of renal fibrosis. Experimental studies confirm that JLD can downregulate the expression of phosphorylated Smad2/3, p-p38 MAPK, and fibronectin (FN) in renal tissues of diabetic kidney disease models (Xue et al., 2013), while upregulating inhibitory Smad7 (Chen L. et al, 2019). This series of molecular events effectively blocks fibroblast activation and abnormal deposition of extracellular matrix, thereby delaying the process of glomerulosclerosis and renal interstitial fibrosis.

Protecting Podocytes, Reducing Proteinuria Leakage: UAER is a key indicator for evaluating the integrity of the glomerular filtration barrier. The excellent reduction of UAER by JLD, especially with greater effect size in patients in the clinical proteinuria stage, highlights its outstanding protective ability for podocytes. Research shows that by activating the AMPK/PGC-1α signaling axis, JLD can fundamentally improve podocyte mitochondrial function, promote mitochondrial fusion, and inhibit excessive division (Sun et al., 2025). Healthy mitochondrial networks ensure energy metabolism and survival of podocytes, thereby significantly inhibiting apoptosis by upregulating Bcl-2 and downregulating Bax and Caspase-3 (Sun et al., 2025). This clearly explains at the cellular molecular level why JLD can effectively reduce urinary protein excretion and maintain the integrity of the filtration barrier.

#### 4.2.2 Systematic metabolic regulation: analyzing synergistic pathways for correcting glucose and lipid metabolism disorders

This study found that JLD can synergistically reduce fasting blood glucose, postprandial blood glucose, glycated hemoglobin, and improve insulin resistance (HOMA-IR) and lipid disorders (TG, TC). This reflects JLD’s source control effect on the initiating factors of DKD—glucolipotoxicity.

Improving Insulin Resistance is the Key Core: The observed reduction in HOMA-IR suggests that improvement of insulin resistance may be one of the key mechanisms through which JLD could exert its hypoglycemic effects. Its mechanism of action is achieved by activating the AMPK/Akt signaling network, the core of energy and metabolic sensing (Zhang, 2014). AMPK, as a key cellular energy sensor, has been proven to improve autophagy activity in diabetes and its complications when activated (Yao et al., 2016). In skeletal muscle, JLD enhances insulin signal transduction, promotes GLUT4 translocation, and increases glucose uptake; in the liver, it inhibits gluconeogenesis and lipid synthesis, promotes fatty acid oxidation; in adipose tissue, it regulates adipokine secretion (such as increasing adiponectin), reduces lipotoxicity and systemic inflammation.

Multi-target Regulation of Adipose Tissue and Pancreatic Islet Function: The regulatory effect of JLD on triglycerides (TG) and total cholesterol (TC), especially more significant in patients with higher baseline TG levels, is closely related to its ability to promote white adipose tissue browning, increase energy consumption, and regulate hepatic lipid metabolism through various mechanisms. Meanwhile, the protective effect of JLD on pancreatic β-cells ensures insulin secretion function, which together with improved insulin resistance constitutes the basis for its comprehensive regulation of glucose homeostasis.

#### 4.2.3 Anti-inflammatory and antioxidant effects: containing the vicious cycle of disease progression

The results of this study suggest that JLD is associated with significant reductions in systemic inflammatory markers such as high-sensitivity C-reactive protein (hs-CRP), tumor necrosis factor-α (TNF-α), and interleukin-6 (IL-6). This indicates that inhibiting micro-inflammatory status is an important pathway for its renal protection.

Inhibiting Core Inflammatory Pathways: The observed decrease in inflammatory factor levels in clinical practice highly coincides with experimental evidence that JLD can inhibit key inflammatory signaling pathways such as NF-κB and TLR4/MyD88 (Deng et al., 2024). It is worth noting that inflammation and fibrosis are the core pathological processes driving DKD progression, involving complex interactions of multiple pathways such as NF-κB, JAK-STAT, and NLRP3 inflammasome (Ansari et al., 2025). The significant reduction of these inflammatory markers by JLD suggests that it may intervene in this complex inflammatory network, blocking the vicious cycle of inflammation-fibrosis from the upstream.

Activating Endogenous Antioxidant Defense: Oxidative stress and inflammation form a vicious cycle, jointly accelerating the progression of DKD. JLD can activate the Nrf2 antioxidant pathway, promote the expression of antioxidant enzymes such as HO-1, and simultaneously enhance mitochondrial function through the FGF21/AMPK pathway (Chen et al., 2023; Tanase et al., 2022). This collectively enhances the body’s ability to clear reactive oxygen species (ROS), reducing oxidative stress damage to renal cells. Additionally, studies have found that JLD can upregulate SIRT1 expression, thereby inhibiting the phosphorylation level of NF-κB p65, which provides another important pathway for its anti-inflammatory mechanism (Lu et al., 2016).

#### 4.2.4 Systems biology perspective of multi-target synergistic effects and clinical value

**FIGURE 9:**
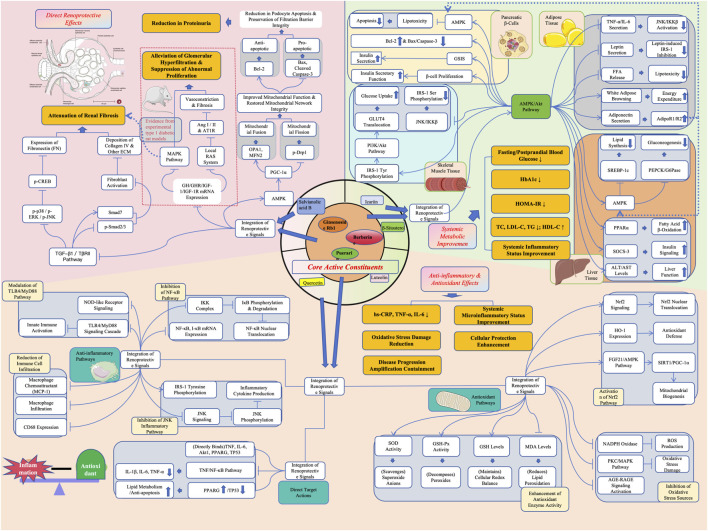
Schematic diagram illustrating the potential mechanisms of Jinlida Granule in the treatment of diabetic kidney disease (DKD). Jinlida Granule acts through multiple pathways: (1) direct renal protection–inhibiting TGF‑β1/Smad signalling to reduce fibrosis, and activating AMPK/PGC‑1α to protect podocytes; (2) metabolic regulation–improving insulin resistance via AMPK/Akt, promoting GLUT4 translocation, and regulating lipid metabolism; (3) anti‑inflammatory and antioxidant effects–suppressing NF‑κB and TLR4/MyD88 pathways, activating Nrf2, and reducing pro‑inflammatory cytokines (TNF‑α, IL‑6, hs‑CRP); and (4) modulation of growth factors (VEGF, IGF‑1). These interconnected mechanisms collectively improve renal function, reduce proteinuria, and delay DKD progression.

If confirmed in more rigorous trials, the multi-dimensional clinical profile of JLD would align with modern DKD treatment concepts from a systems biology perspective. In recent years, guideline-directed medical therapy (GDMT) emphasizes maximizing delay of DKD progression through multi-drug combination strategies (Madero et al., 2025; Wanner et al., 2025). The theoretical basis for this combination strategy lies in the fact that DKD itself is a complex disease involving multiple mechanisms and pathways, and single-target interventions are often difficult to effectively curb disease progression (DeFronzo et al., 2021). Jinlida Granule, as a multi-botanical preparation, its “multi-metabolite- multi-target- multi-pathway” action characteristics essentially reflect a “built-in combination therapy strategy.” Network pharmacology studies depict a clear blueprint of action: its core active components can jointly act on key targets such as TNF, AKT1, IL-6, PPARG, which are significantly enriched in signaling pathways closely related to DKD such as AMPK, insulin resistance, TNF, MAPK (Su et al., 2025). This systematic level of network regulation enables it to simultaneously intervene in multiple pathological processes such as metabolism, inflammation, and fibrosis (Ansari et al., 2025; Chen et al., 2021). Importantly, as DeFronzo et al. (2021) pointed out, although SGLT2 inhibitors can improve various pathophysiological abnormalities in DKD, since multiple pathophysiological disorders jointly contribute to the occurrence and progression of DKD, it is likely that multiple drugs need to be used in combination to effectively delay disease progression. This further supports the rationality and advantage of JLD as a multi-target therapeutic botanical preparation. Different from the modern drug combination therapy concept, JLD achieves synergistic regulation of multiple key pathological links through a single compound (Liu et al., 2023; Wang et al., 2023), providing a unique and promising option for adjuvant therapy of DKD. This systematic level of network regulation enables Jinlida Granule to simultaneously intervene in multiple pathological processes such as metabolism, inflammation, and fibrosis. A comprehensive schematic diagram summarizing these multi-target mechanisms of Jinlida Granule in the treatment of diabetic kidney disease is presented in Figure 9.

### 4.3 Strengths and Limitations

The strengths of this systematic review lie in its comprehensiveness and depth. It not only comprehensively evaluated the multi-dimensional effects of Jinlida Granule on DKD renal function, glucose and lipid metabolism, and micro-inflammatory status for the first time, but also deeply analyzed the sources of efficacy heterogeneity through pre-specified, clinically practice-based subgroup analyses, identifying patient populations and combination strategies that may benefit the most, thus advancing the evidence level from “whether it is effective” to “for whom it is more effective”.

However, this study also has several limitations. The primary issue is that the methodological quality of the included original studies is mostly moderate, generally lacking detailed descriptions of allocation concealment and blinding implementation, which may introduce potential performance and measurement biases. Secondly, although the treatment duration is sufficient to assess changes in intermediate biomarkers, long-term follow-up data are lacking to evaluate its impact on hard endpoints such as end-stage renal disease and cardiovascular events. For example, among the studies included in this analysis, only a few reported ESRD incidence or cardiovascular events (Wang, 2023), while most studies used surrogate endpoints such as serum creatinine and urinary protein as primary outcomes (Su, 2020). Third, meta-regression was planned to explore potential sources of heterogeneity, but the number of included studies for each outcome was insufficient to support reliable meta-regression analysis. For the primary outcome (Scr), exploratory meta-regression showed that combination regimen type did not explain the observed heterogeneity (P = 0.881, Adj *R* ^2^ = −17.52%), likely due to the limited number of studies (n = 8) and the complex, multi-factorial nature of the heterogeneity. For UAER, meta-regression was not feasible because of insufficient variation in the covariate. For other outcomes, the number of studies was below the recommended threshold of 10 per covariate (Higgins et al., 2023). This limitation further underscores the need for larger, well-reported trials to enable robust exploration of effect modifiers. Fourth, the reporting of adverse events was inconsistent across the included studies. Two studies did not report safety data, and several others described adverse events only qualitatively without providing specific incidence figures. Future trials should adopt standardized frameworks for adverse event monitoring and reporting to enable more robust safety evaluation.

### 4.4 Implications for future research and clinical practice

Based on the above findings and limitations, future research should be dedicated to conducting well-designed, large-sample, multi-center, and long-term follow-up randomized controlled trials to confirm the protective effects of Jinlida Granule on renal and cardiovascular hard endpoints. It is worth noting that the current frontier of DKD pharmacological treatment has entered an exploration stage of ‘strong combination of pillar drugs’. For example, the benefits and safety of the combination therapy of Finerenone and SGLT2i have become the focus of systematic reviews (Wang et al., 2025). The studies by Xia et al. (2025) and Deng et al. (2020) included in this analysis provide preliminary positive signals for the synergistic benefits of combining Jinlida Granule with new hypoglycemic drugs such as SGLT2 inhibitors or GLP-1 receptor agonists, which deserve further validation in larger-scale trials. At the basic research level, omics technologies and molecular biology methods should be fully utilized to deeply elucidate the specific molecular mechanisms of its multi-component, multi-target, multi-pathway synergistic effects (Su et al., 2025; Wang, 2023).

In clinical practice, this study provides evidence-based support for adding Jinlida Granule on the basis of standard biomedicine treatment, especially for early DKD patients, to seek additional renal and metabolic benefits. When applying it, clinicians and pharmacists can refer to the latest guideline spirit (Awdishu et al., 2025; Madero et al., 2025), comprehensively consider the patient’s specific stage, comorbidities, and treatment plan, to achieve individualized treatment decisions (Awdishu et al., 2025).

## 5 Conclusion

In conclusion, this systematic review and meta-analysis indicates that adjunctive use of Jinlida Granule with conventional therapy may offer additional clinical benefits in terms of renoprotection, metabolic regulation, and anti-inflammatory effects for DKD patients. Subgroup analyses further suggest that patients with early-stage DKD receiving a “Jinlida Granule combined with basic biomedicine treatment” regimen may obtain more stable renal protection benefits. However, the overall certainty of evidence is limited by the moderate to high risk of bias in the included trials and substantial unexplained heterogeneity across most outcomes. These findings should therefore be interpreted with caution. Well-designed, large-scale RCTs with long-term follow-up are required to confirm the efficacy and safety of JLD and to evaluate its impact on hard clinical endpoints.

## Data Availability

The original contributions presented in the study are included in the article/Supplementary Material, further inquiries can be directed to the corresponding author.
