# Supplementary material for: Efficacy and safety of jinlida granule in the treatment of diabetic kidney disease: a systematic review and meta-analysis of randomized controlled trials
Source: Front Pharmacol. 2026 Jun 3;17:1761454. doi: 10.3389/fphar.2026.1761454 (PMC13272490; doi:10.3389/fphar.2026.1761454)
Supplement: Supplementary file 1 [file Supplementaryfile1.pdf]

Supplementary Material

Contents Page

1 Supplementary Material S1. PRISMA 2020 checklist 3
2 Supplementary Material S2. Database and Search Strategies 9
2.1 PubMed 9
2.2 Embase 9
2.3 Cochrane 10
2.4 WOS 12
2.5 CNKI 13
2.6 Wan Fang 13
2.7 VIP 14
2.8 CBM 15
2.9 ClinicalTrials.gov 16
2.10 Chinese Clinical Trial Registry 16
3 Supplementary Material S3. Graphical abstract of Jinlida_DKD. 18
4 Supplementary Material S4. Detailed Risk of Bias Assessment 20
5 Supplementary Material S5. Botanical Information of Jinlida Granule Composition 25
5.1 Part 1: Botanical Information of Jinlida Granule Composition 25
5.2 Part 2: Chemical Analysis Evidence for Jinlida Granule Quality Control 26
6 Supplementary Material S6. Subgroup analysis 28
6.1 Subgroup analysis for renal function indicators 28
6.1.1 Forest Plots for Subgroup Analysis of Renal Function Indicators29
6.1.2 Table renal content： Subgroup analysis for Scr, BUN, UAER, and 24hUP 30
6.2 Subgroup analysis for glucose and lipid metabolism indicators 32
6.2.1 Forest Plots for Subgroup Analysis of Glucose and Lipid Metabolism Indicators 32
6.2.2 Table glucose content: Subgroup analysis for FBG, 2hPG, HbA1c, TG, and TC 32
6.3 Subgroup Analysis for Inflammatory Markers 35
6.3.1 Forest Plots for Subgroup Analysis of Inflammatory Markers 36

6.3.2 Table inflammatory content: Subgroup analysis for hs-CRP, TNF-α, and IL-6 37

6.4 Subgroup Analysis for Growth Factors 39

6.4.1 Forest Plots for Subgroup Analysis of Growth Factors 39

6.4.2 Table growth content: Subgroup analysis for VEGF and IGF-1 . 40

7 Supplementary Material S7. Sensitivity analysis 41

7.1 Supplementary Figure : Sensitivity analysis 41

7.2 Supplementary Table : Sensitivity Analysis Results 42

8 Supplementary Material S8.Traditional Use and Ethnopharmacological Relevance Documentation 44

8.1 Traditional Chinese Medicine Theoretical Foundation 44

8.2 Jinlida Granule Formula Principles and Composition Rationale 45

8.3 Modern Scientific Evidence Supporting Traditional Use 46

8.4 Integration with Contemporary DKD Pathophysiology 47

9 Supplementary Material S10. Statistical Analysis Code - STATA Commands 48

1 Supplementary Material S1. PRISMA 2020 checklist

Section and Topic	Item #	Checklist item	Location where item is reported
**TITLE**			
Title	1	Identify the report as a systematic review.	*Title page*
**ABSTRACT**			
Abstract	2	See the PRISMA 2020 for Abstracts checklist.	*Abstract section*
**INTRODUCTION**			
Rationale	3	Describe the rationale for the review in the context of existing knowledge.	
Objectives	4	Provide an explicit statement of the objective(s) or question(s) the review addresses.	
**METHODS**			
Eligibility criteria	5	Specify the inclusion and exclusion criteria for the review and how studies were grouped for the syntheses.	

Information sources	6	Specify all databases, registers, websites, organisations, reference lists and other sources searched or consulted to identify studies. Specify the date when each source was last searched or consulted.	***(search date: October 9, 2025)***
Search strategy	7	Present the full search strategies for all databases, registers and websites, including any filters and limits used.	Supplementary Material S2
Selection process	8	Specify the methods used to decide whether a study met the inclusion criteria of the review, including how many reviewers screened each record and each report retrieved, whether they worked independently, and if applicable, details of automation tools used in the process.	
Data collection process	9	Specify the methods used to collect data from reports, including how many reviewers collected data from each report, whether they worked independently, any processes for obtaining or confirming data from study investigators, and if applicable, details of automation tools used in the process.	
Data items	10a	List and define all outcomes for which data were sought. Specify whether all results that were compatible with each outcome domain in each study were sought (e.g. for all measures, time points, analyses), and if not, the methods used to decide which results to collect.	
	10b	List and define all other variables for which data were sought (e.g. participant and intervention characteristics, funding sources). Describe any assumptions made about any missing or unclear information.	
Study risk of bias assessment	11	Specify the methods used to assess risk of bias in the included studies, including details of the tool(s) used, how many reviewers assessed each study and whether they worked independently, and if applicable, details of automation tools used in the process.	

Effect measures	12	Specify for each outcome the effect measure(s) (e.g. risk ratio, mean difference) used in the synthesis or presentation of results.	
Synthesis methods	13a	Describe the processes used to decide which studies were eligible for each synthesis (e.g. tabulating the study intervention characteristics and comparing against the planned groups for each synthesis (item #5)).	
	13b	Describe any methods required to prepare the data for presentation or synthesis, such as handling of missing summary statistics, or data conversions.	
	13c	Describe any methods used to tabulate or visually display results of individual studies and syntheses.	
	13d	Describe any methods used to synthesize results and provide a rationale for the choice(s). If meta-analysis was performed, describe the model(s), method(s) to identify the presence and extent of statistical heterogeneity, and software package(s) used.	
	13e	Describe any methods used to explore possible causes of heterogeneity among study results (e.g. subgroup analysis, meta-regression).	
	13f	Describe any sensitivity analyses conducted to assess robustness of the synthesized results.	
Reporting bias assessment	14	Describe any methods used to assess risk of bias due to missing results in a synthesis (arising from reporting biases).	
Certainty assessment	15	Describe any methods used to assess certainty (or confidence) in the body of evidence for an outcome.	

RESULTS			
Study selection	16a	Describe the results of the search and selection process, from the number of records identified in the search to the number of studies included in the review, ideally using a flow diagram.	*Figure 1*
	16b	Cite studies that might appear to meet the inclusion criteria, but which were excluded, and explain why they were excluded.	*Supplementary Material S11*
Study characteristics	17	Cite each included study and present its characteristics.	*Table 1, Supplementary Material S5*
Risk of bias in studies	18	Present assessments of risk of bias for each included study.	Figure 2
Results of individual studies	19	For all outcomes, present, for each study: (a) summary statistics for each group (where appropriate) and (b) an effect estimate and its precision (e.g. confidence/credible interval), ideally using structured tables or plots.	
Results of syntheses	20a	For each synthesis, briefly summarise the characteristics and risk of bias among contributing studies.	
	20b	Present results of all statistical syntheses conducted. If meta-analysis was done, present for each the summary estimate and its precision (e.g. confidence/credible interval) and measures of statistical heterogeneity. If comparing groups, describe the direction of the effect.	
	20c	Present results of all investigations of possible causes of heterogeneity among study results.	

	20d	Present results of all sensitivity analyses conducted to assess the robustness of the synthesized results.	
Reporting biases	21	Present assessments of risk of bias due to missing results (arising from reporting biases) for each synthesis assessed.	
Certainty of evidence	22	Present assessments of certainty (or confidence) in the body of evidence for each outcome assessed.	Supplementary Material S11
**DISCUSSION**			
Discussion	23a	Provide a general interpretation of the results in the context of other evidence.	
	23b	Discuss any limitations of the evidence included in the review.	
	23c	Discuss any limitations of the review processes used.	
	23d	Discuss implications of the results for practice, policy, and future research.	
**OTHER INFORMATION**			
Registration and protocol	24a	Provide registration information for the review, including register name and registration number, or state that the review was not registered.	*P4, CRD420251178318*
	24b	Indicate where the review protocol can be accessed, or state that a protocol was not prepared.	-
	24c	Describe and explain any amendments to information provided at registration or in the protocol.	-

Support	25	Describe sources of financial or non-financial support for the review, and the role of the funders or sponsors in the review.	
Competing interests	26	Declare any competing interests of review authors.	
Availability of data, code and other materials	27	Report which of the following are publicly available and where they can be found: template data collection forms; data extracted from included studies; data used for all analyses; analytic code; any other materials used in the review.	

From: Page MJ, McKenzie JE, Bossuyt PM, Boutron I, Hoffmann TC, Mulrow CD, et al. The PRISMA 2020 statement: an updated guideline for reporting systematic reviews. BMJ 2021;372:n71. doi: 10.1136/bmj.n71

2 Supplementary Material S2. Database and Search Strategies

2.1 PubMed

The retrieval of the PubMed database was conducted on October 9, 2025, and no records were retrieved.

Search	Query	Results
#6	((#3) AND (#4)) AND (#5)	0
#5	"randomized controlled trial"[Title/Abstract] OR "randomised controlled trial"[Title/Abstract] OR "RCT"[Title/Abstract] OR "randomly"[Title/Abstract] OR "randomized"[Title/Abstract] OR "randomised"[Title/Abstract] OR "placebo"[Title/Abstract] OR "trial"[Title/Abstract] OR "clinical trial"[Title/Abstract] OR "controlled clinical trial"[Title/Abstract]	1785187
#4	"Jinlida"[Title/Abstract] OR "JLD"[Title/Abstract] OR "Jinlida Granule"[Title/Abstract] OR "Jinlida Granules"[Title/Abstract]	37
#3	(#1) OR (#2)	45020
#2	(Nephropathies, Diabetic [Title/Abstract] OR Nephropathy, Diabetic [Title/Abstract] OR Diabetic Kidney Disease [Title/Abstract] OR Diabetic Kidney Diseases [Title/Abstract] OR Kidney Disease, Diabetic [Title/Abstract] OR Kidney Diseases, Diabetic [Title/Abstract] OR Diabetic Nephropathy [Title/Abstract] OR Diabetic Glomerulosclerosis [Title/Abstract] OR Glomerulosclerosis, Diabetic [Title/Abstract] OR Intracapillary Glomerulosclerosis [Title/Abstract] OR Kimmelstiel - Wilson Disease [Title/Abstract] OR Kimmelstiel Wilson Disease [Title/Abstract] OR Nodular Glomerulosclerosis [Title/Abstract] OR Glomerulosclerosis, Nodular [Title/Abstract] OR Kimmelstiel - Wilson Syndrome [Title/Abstract] OR Kimmelstiel Wilson Syndrome [Title/Abstract] OR Syndrome, Kimmelstiel - Wilson [Title/Abstract])	32129
#1	"Diabetic Nephropathies"[Mesh]	32563

2.2 Embase

The retrieval of the Embase database was conducted on October 9, 2025, and no records were retrieved.

Search		Results
#6	#3 AND #4 AND #5	0
#5	'rct':ti,ab,kw OR 'randomly':ti,ab,kw OR 'randomized':ti,ab,kw OR 'randomised':ti,ab,kw OR 'placebo':ti,ab,kw OR 'trial':ti,ab,kw OR 'clinical trial':ti,ab,kw OR 'controlled clinical trial':ti,ab,kw OR 'diabetic	2860220

	kidney disease':ti,ab,kw	
#4	'jinlida':ti,ab,kw OR 'jin li da':ti,ab,kw OR 'jin-li-da':ti,ab,kw OR 'jld':ti,ab,kw OR 'jinlida granule':ti,ab,kw OR 'jinlida granules':ti,ab,kw OR 'jinlida keli':ti,ab,kw OR 'jinlida capsule':ti,ab,kw OR 'jinlida capsules':ti,ab,kw OR 'jinlida tablet':ti,ab,kw OR 'jinlida tablets':ti,ab,kw OR 'jinlida oral liquid':ti,ab,kw OR 'jinlida koufuye':ti,ab,kw OR 'jinlida pill':ti,ab,kw OR 'jinlida pills':ti,ab,kw OR 'jinlida powder':ti,ab,kw OR 'jinlida san':ti,ab,kw	133
#3	#1 OR #2	73715
#2	'nephropathies, diabetic':ti,ab,kw OR 'nephropathy, diabetic':ti,ab,kw OR 'diabetic kidney diseases':ti,ab,kw OR 'kidney disease, diabetic':ti,ab,kw OR 'kidney diseases, diabetic':ti,ab,kw OR 'diabetic nephropathy':ti,ab,kw OR 'diabetic glomerulosclerosis':ti,ab,kw OR 'glomerulosclerosis, diabetic':ti,ab,kw OR 'intracapillary glomerulosclerosis':ti,ab,kw OR 'kimmelstiel-wilson disease':ti,ab,kw OR 'kimmelstiel wilson disease':ti,ab,kw OR 'nodular glomerulosclerosis':ti,ab,kw OR 'glomerulosclerosis, nodular':ti,ab,kw OR 'kimmelstiel-wilson syndrome':ti,ab,kw OR 'kimmelstiel wilson syndrome':ti,ab,kw OR 'syndrome, kimmelstiel-wilson':ti,ab,kw OR 'diabetic kidney disease':ti,ab,kw	48051
#1	'diabetic nephropathy' exp OR 'diabetic nephropathy'	71935

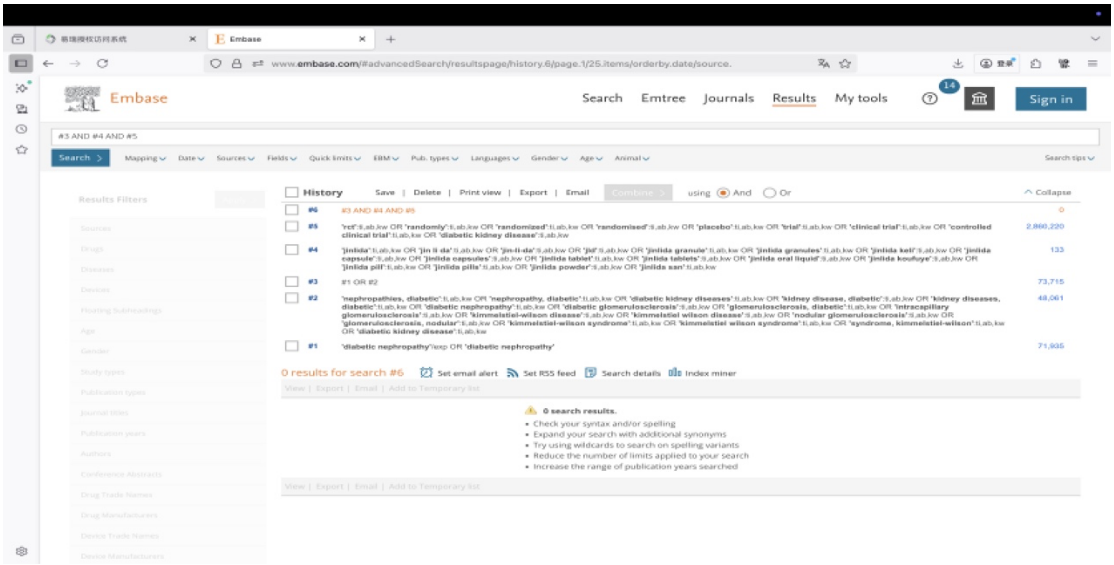

2.3 Cochrane

The retrieval of the Cochrane Library was conducted on October 9, 2025, and no records were retrieved.

Search		Results
#5	#3 AND #4	1
#4	'jinlida':ti,ab,kw OR 'jin li da':ti,ab,kw OR 'jin-li-da':ti,ab,kw OR 'jld':ti,ab,kw OR 'jinlida granule':ti,ab,kw OR 'jinlida granules':ti,ab,kw OR 'jinlida keli':ti,ab,kw OR 'jinlida capsule':ti,ab,kw OR 'jinlida capsules':ti,ab,kw OR 'jinlida tablet':ti,ab,kw OR 'jinlida tablets':ti,ab,kw OR 'jinlida oral liquid':ti,ab,kw OR 'jinlida koufuye':ti,ab,kw OR 'jinlida pill':ti,ab,kw OR 'jinlida pills':ti,ab,kw OR 'jinlida powder':ti,ab,kw OR 'jinlida san':ti,ab,kw	45
#3	#1 OR #2	7611
#2	'nephropathies, diabetic':ti,ab,kw OR 'nephropathy, diabetic':ti,ab,kw OR 'diabetic kidney diseases':ti,ab,kw OR 'kidney disease, diabetic':ti,ab,kw OR 'kidney diseases, diabetic':ti,ab,kw OR 'diabetic nephropathy':ti,ab,kw OR 'diabetic glomerulosclerosis':ti,ab,kw OR 'glomerulosclerosis, diabetic':ti,ab,kw OR 'intracapillary glomerulosclerosis':ti,ab,kw OR 'kimmelstiel-wilson disease':ti,ab,kw OR 'kimmelstiel wilson disease':ti,ab,kw OR 'nodular glomerulosclerosis':ti,ab,kw OR 'glomerulosclerosis, nodular':ti,ab,kw OR 'kimmelstiel-wilson syndrome':ti,ab,kw OR 'kimmelstiel wilson syndrome':ti,ab,kw OR'syndrome, kimmelstiel-wilson':ti,ab,kw OR 'diabetic kidney disease':ti,ab,kw	7611
#1	MeSH descriptor: [Diabetic Nephropathies] explode all trees	2046

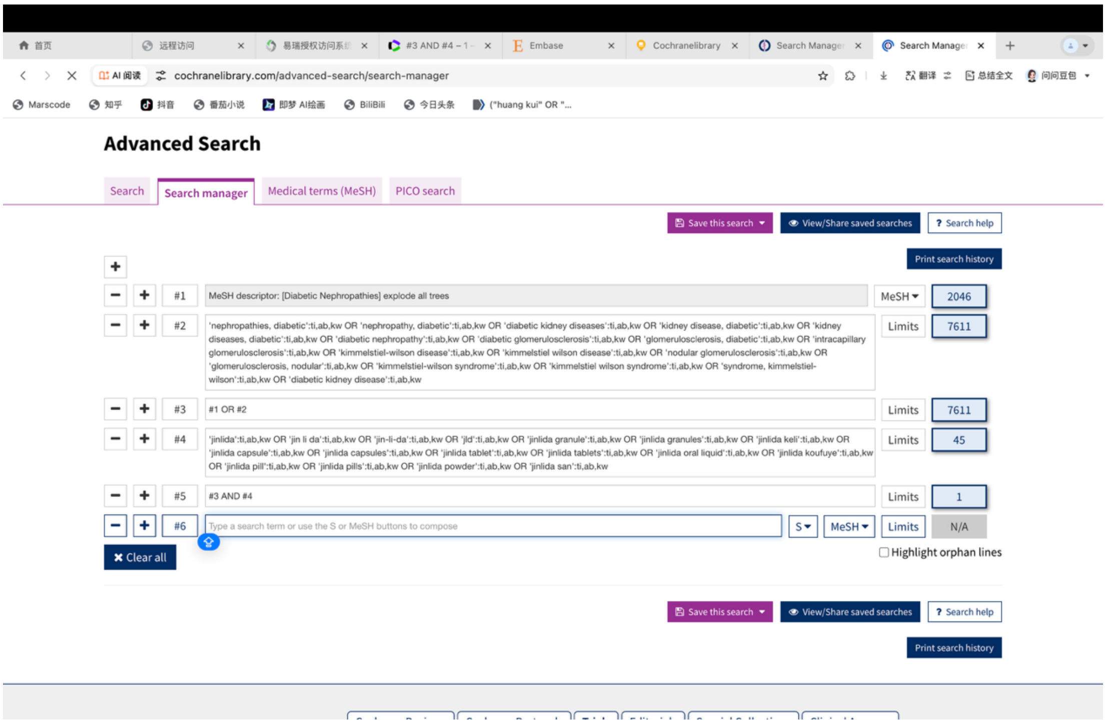

2.4 WOS

The retrieval of the WOS database was conducted on October 9, 2025, and no records were retrieved.

History	Search Query	Results
#6	#3 AND #4 AND #5	0
#5	TS=(("randomized controlled trial" OR "randomised controlled trial" OR "RCT" OR "randomly" OR "randomized" OR "randomised" OR "placebo" OR "trial" OR "clinical trial" OR "controlled clinical trial" OR "diabetic kidney disease"))	1515784
#4	TS=(("Jinlida") OR ("Jin Li Da") OR ("Jin-Li-Da") OR ("JLD") OR ("Jinlida Granule") OR ("Jinlida Granules") OR ("Jinlida Keli") OR ("Jinlida Capsule") OR ("Jinlida Capsules") OR ("Jinlida Tablet") OR ("Jinlida Tablets") OR ("Jinlida Oral Liquid") OR ("Jinlida Koufuye") OR ("Jinlida Pill") OR ("Jinlida Pills") OR ("Jinlida Powder") OR ("Jinlida San"))	82
#3	#2 OR #1	28644
#2	TS = (("Nephropathies, Diabetic" OR "Nephropathy, Diabetic" OR "Diabetic Kidney Disease" OR "Diabetic Kidney Diseases" OR "Kidney Disease, Diabetic" OR "Kidney Diseases, Diabetic" OR "Diabetic Nephropathy" OR "Diabetic Glomerulosclerosis" OR "Glomerulosclerosis, Diabetic" OR "Intracapillary Glomerulosclerosis" OR "Kimmelstiel-Wilson Disease" OR "Kimmelstiel Wilson Disease" OR "Nodular Glomerulosclerosis" OR "Glomerulosclerosis, Nodular" OR "Kimmelstiel-Wilson Syndrome" OR "Kimmelstiel Wilson Syndrome" OR "Syndrome, Kimmelstiel-Wilson"))	25449
#1	diabetic nephropathy (Topic)	25282

2.5 CNKI

The retrieval of the CNKI database was conducted on October 9, 2025, and a total of 18 records were retrieved.

(SU=’津力达’+’津力达颗粒’+’金力达’+’津力达胶囊’+’Jinlida’+’津力达口服液’+’津力达浓缩丸’+’津力达丸’ OR TKA=’津力达’+’津力达颗粒’+’金力达’+’津力达胶囊’+’Jinlida’+’津力达口服液’+’津力达浓缩丸’+’津力达丸’)

AND

(SU=’糖尿病肾病’+’糖尿病肾脏疾病’+’糖尿病肾小球硬化症’+’糖尿病性肾病’+’糖尿病性肾脏病’+’DN’+’DKD’+’糖尿病肾病期’+’糖尿病肾损害’+’糖尿病肾并发症’ OR TKA=’糖尿病肾病’+’糖尿病肾脏疾病’+’糖尿病肾小球硬化症’+’糖尿病性肾病’+’糖尿病性肾脏病’+’DN’+’DKD’+’糖尿病肾病期’+’糖尿病肾损害’+’糖尿病肾并发症’)

AND

(AB=’随机对照试验’+’随机对照研究’+’随机临床试验’+’RCT’+’随机分配’+’随机’+’随机方法’+’对照试验’+’临床随机试验’)

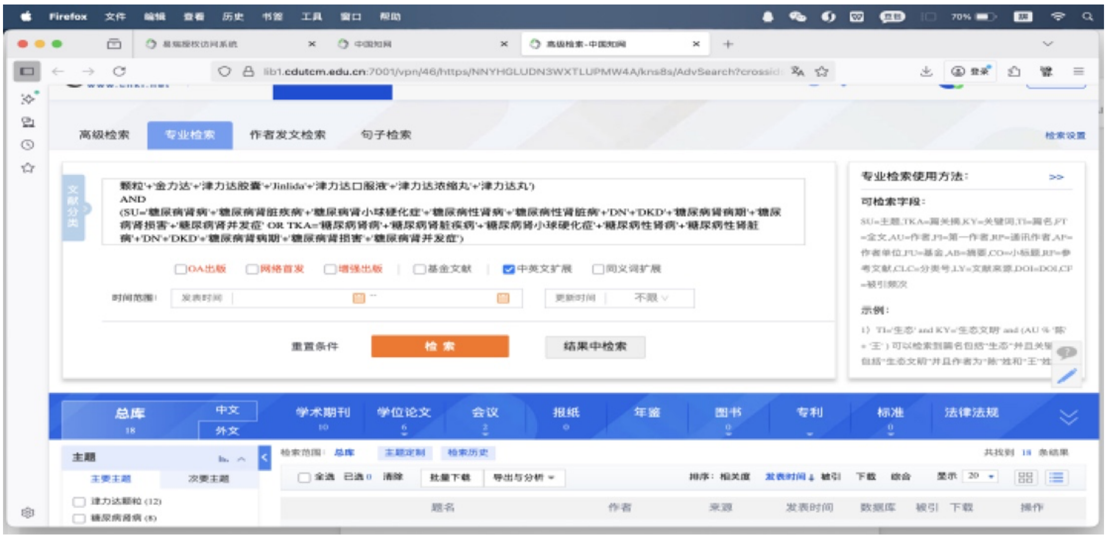

2.6 Wan Fang

The retrieval of the Wan Fang database was conducted on October 9, 2025, and a total of 51 records were retrieved.

(主题:(津力达 or 津力达颗粒 or 金力达 or 津力达胶囊 or Jinlida or 津力达口服液 or 津力达浓缩丸 or 津力达丸) or 题名或关键词:(津力达 or 津力达颗粒 or 金力达 or 津力达胶囊 or Jinlida or 津力达口服液 or 津力达

浓缩丸 or 津力达丸)) and (主题:(糖尿病肾病 or 糖尿病肾脏疾病 or 糖尿病肾小球硬化症 or 糖尿病性肾病 or 糖尿病性肾脏病 or DN or DKD or 糖尿病肾病期 or 糖尿病肾损害 or 糖尿病肾并发症) or 题名或关键词:(糖尿病肾病 or 糖尿病肾脏疾病 or 糖尿病肾小球硬化症 or 糖尿病性肾病 or 糖尿病性肾脏病 or DN or DKD or 糖尿病肾病期 or 糖尿病肾损害 or 糖尿病肾并发症)) and (摘要:(随机对照试验 or 随机对照研究 or 随机临床试验 or RCT or 随机分配 or 随机 or 随机方法 or 对照试验 or 临床随机试验))

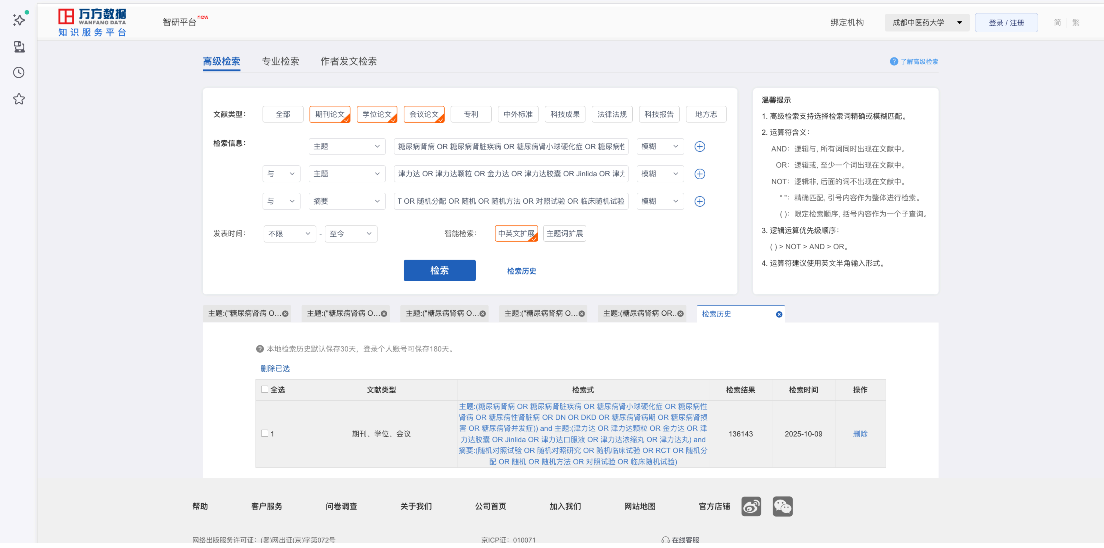

2.7 VIP

The retrieval of the VIP database was conducted on October 9, 2025, and a total of 18 records were retrieved.

((M=(津力达+津力达颗粒+金力达+津力达胶囊+Jinlida+津力达口服液+津力达浓缩丸+津力达丸)) OR (R=(津力达+津力达颗粒+金力达+津力达胶囊+Jinlida+津力达口服液+津力达浓缩丸+津力达丸))) AND ((M=(糖尿病肾病+糖尿病肾脏疾病+糖尿病肾小球硬化症+糖尿病性肾病+糖尿病性肾脏病+DN+DKD+糖尿病肾病期+糖尿病肾损害+糖尿病肾并发症)) OR (R=(糖尿病肾病+糖尿病肾脏疾病+糖尿病肾小球硬化症+糖尿病性肾病+糖尿病性肾脏病+DN+DKD+糖尿病肾病期+糖尿病肾损害+糖尿病肾并发症))) AND (R=(随机对照试验+随机对照研究+随机临床试验+RCT+随机分配+随机+随机方法+对照试验+临床随机试验))

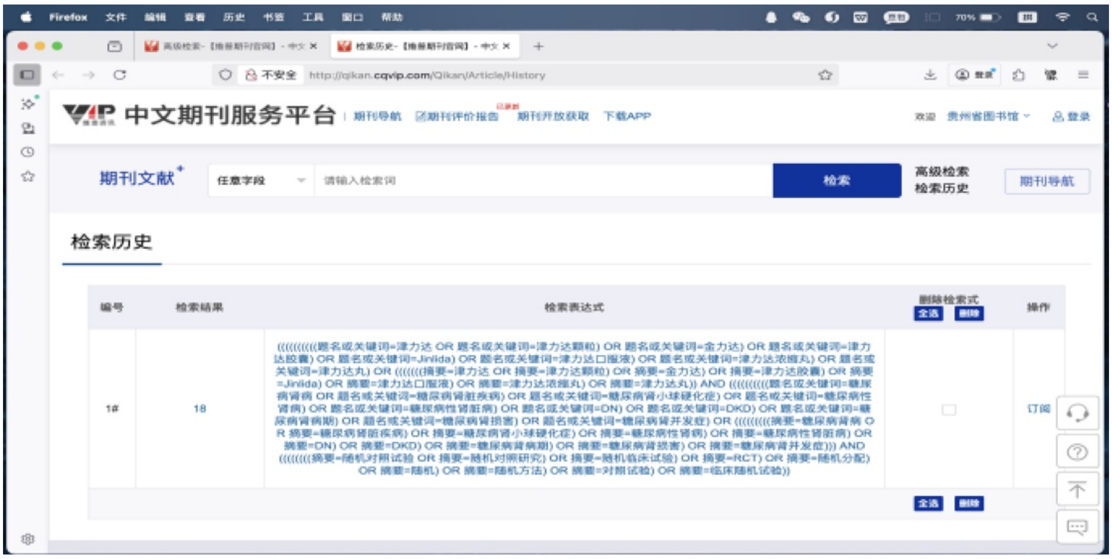

2.8 CBM

The retrieval of the CBM database was conducted on October 9, 2025, and a total of 13 records were retrieved.

（（（"糖尿病肾病"[不加权:扩展]）OR（"糖尿病肾脏疾病"[常用字段:智能] OR "糖尿病肾小球硬化症"[常用字段:智能] OR "糖尿病性肾病"[常用字段:智能] OR "糖尿病性肾脏病"[常用字段:智能] OR "DN"[常用字段:智能] OR "DKD"[常用字段:智能] OR "糖尿病肾病期"[常用字段:智能] OR "糖尿病肾损害"[常用字段:智能] OR "糖尿病肾并发症"[常用字段:智能]））AND（（"津力达颗粒"[不加权:扩展]）OR（"津力达"[常用字段:智能] OR "金力达"[常用字段:智能] OR "津力达胶囊"[常用字段:智能] OR "Jinlida"[常用字段:智能] OR "津力达口服液"[常用字段:智能] OR "津力达浓缩丸"[常用字段:智能] OR "津力达丸"[常用字段:智能]））AND（ "随机对照试验"[摘要] OR "随机对照研究"[摘要] OR "随机临床试验"[摘要] OR "RCT"[摘要] OR "随机分配"[摘要] OR "随机"[摘要] OR "随机方法"[摘要] OR "对照试验"[摘要] OR "临床随机试验"[摘要]））

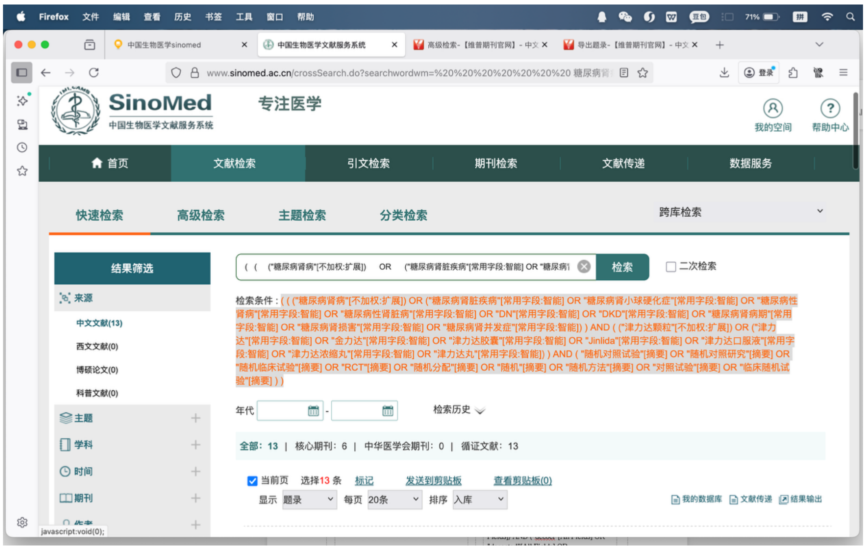

2.9 ClinicalTrials.gov

The retrieval of the ClinicalTrials.gov Registry was conducted on October 9, 2025, and no records were retrieved.

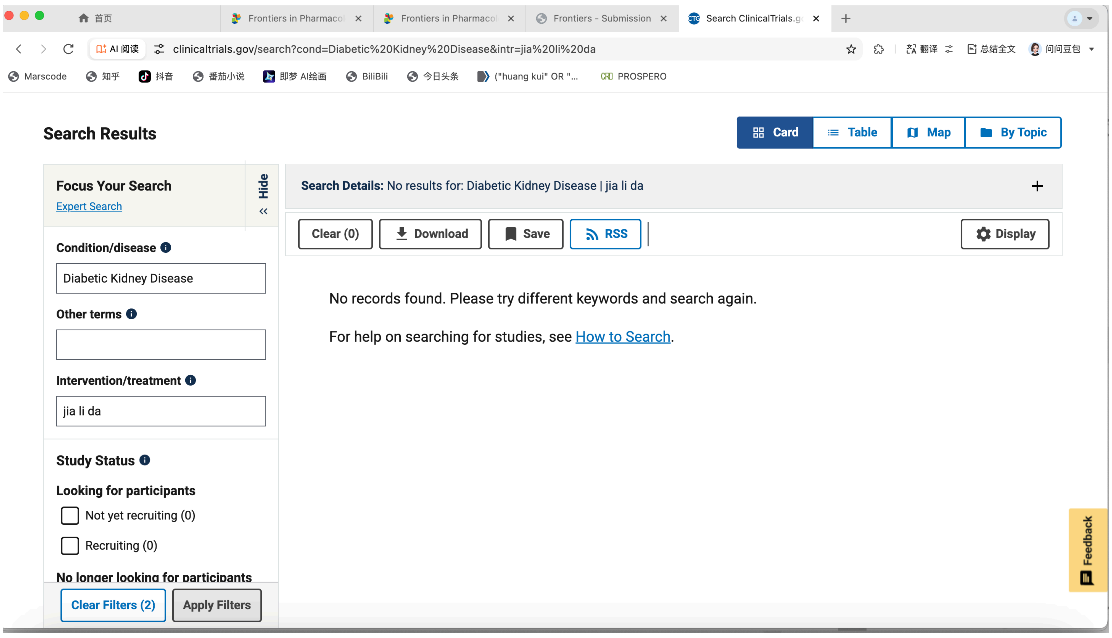

2.10 Chinese Clinical Trial Registry

The retrieval of the Chinese Clinical Trial Registry was conducted on October 9, 2025, and no records were retrieved.

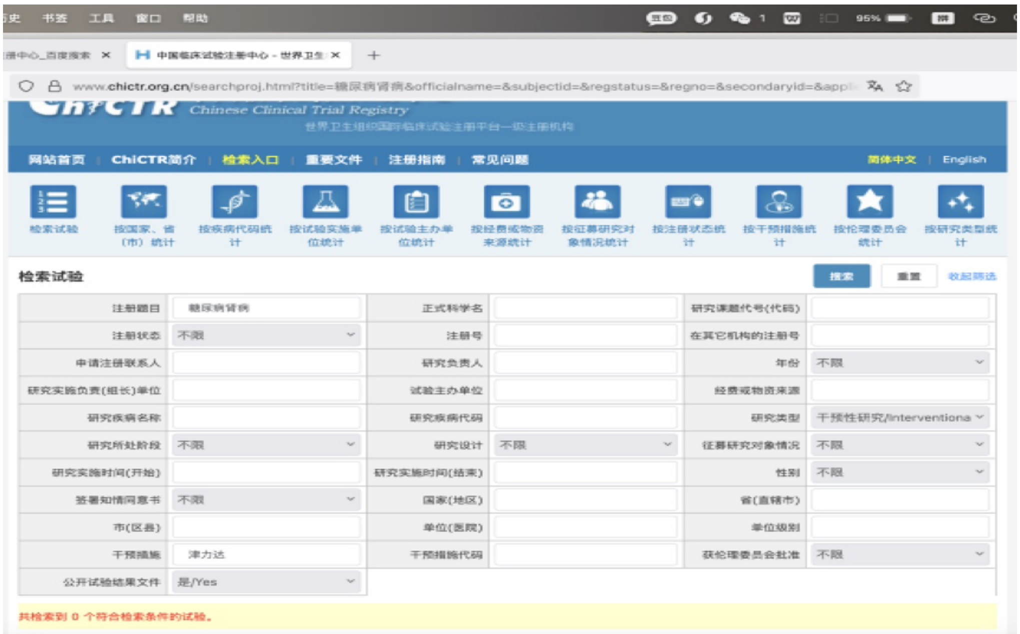
书签
工具
窗口
帮助
95%
中国临床试验注册中心 - 世界卫生
www.chictr.org.cn/searchproj.html?title=糖尿病肾病&officialname=&subjectid=&regstatus=&regno=&secondaryid=&appl
Chinese Clinical Trial Registry
世界卫生组织国际临床试验注册平台一级注册机构
网站首页
ChiCTR简介
检索入口
重要文件
注册指南
常见问题
简体中文
English
检索试验
按国家、省（市）统计
按疾病代码统计
按试验实施单位统计
按试验主办单位统计
按经费或物资来源统计
按征募研究对象情况统计
按注册状态统计
按干预措施统计
按伦理委员会统计
按研究类型统计
检索试验
搜索
重置
收起筛选
注册题目
糖尿病肾病
正式科学名
研究课题代号(代码)
注册状态
不限
注册号
在其它机构的注册号
申请注册联系人
研究负责人
年份
不限
研究实施负责(组长)单位
试验主办单位
经费或物资来源
研究疾病名称
研究疾病代码
研究类型
干预性研究/Interventiona
研究所处阶段
不限
研究设计
不限
征募研究对象情况
不限
研究实施时间(开始)
研究实施时间(结束)
性别
不限
签署知情同意书
不限
国家(地区)
省(直辖市)
市(区县)
单位(医院)
单位级别
干预措施
津力达
干预措施代码
获伦理委员会批准
不限
公开试验结果文件
是/Yes
共检索到 0 个符合检索条件的试验。

3 Supplementary Material S3. Graphical abstract of Jinlida_DKD.

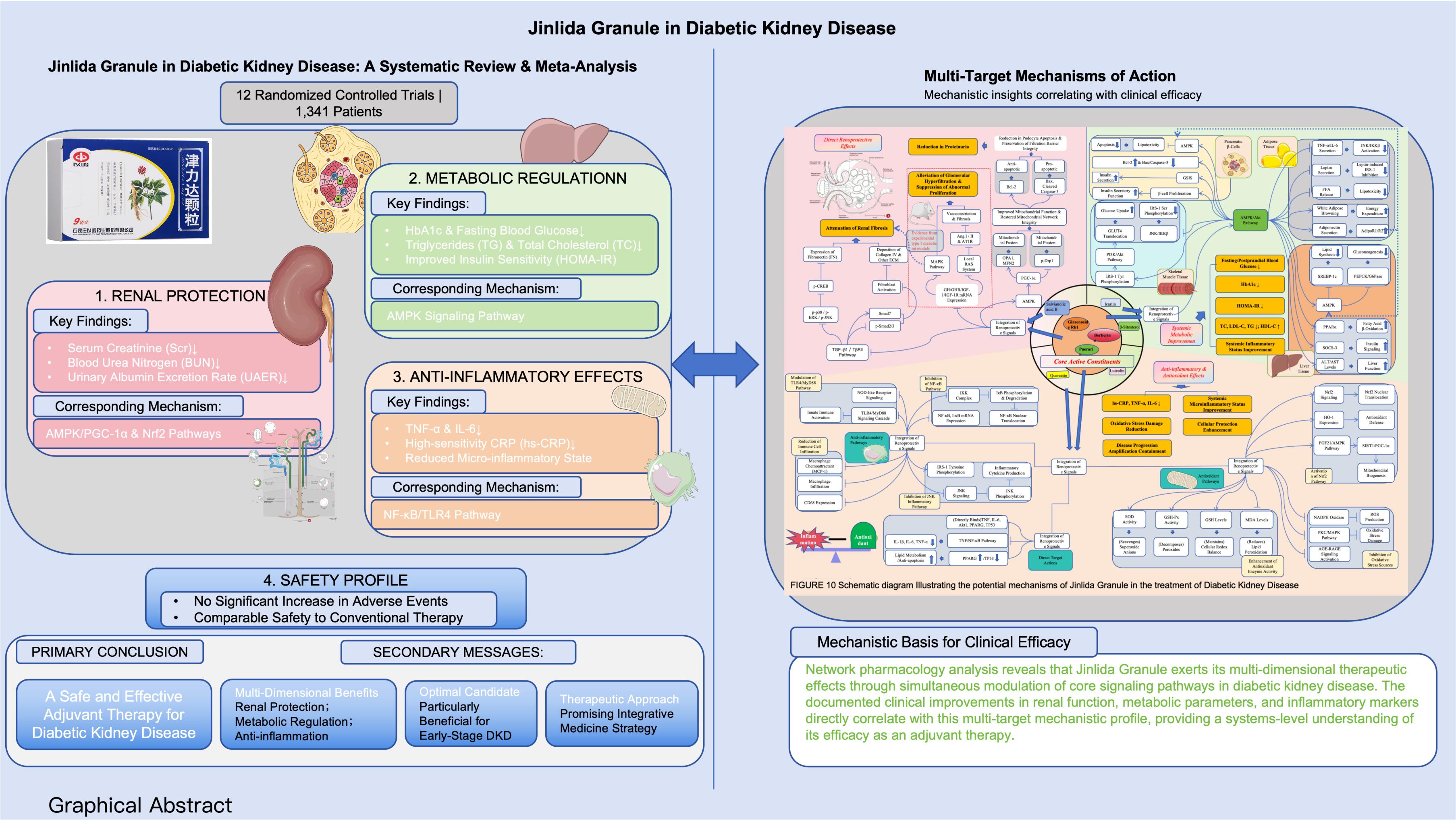

Description:

This graphical abstract provides a visual synthesis of the systematic review and meta-analysis evaluating Jinlida Granule (JLD) as an adjunctive therapy for Diabetic Kidney Disease (DKD). It integrates the key clinical findings from 12 randomized controlled trials with the underlying multi-target mechanisms of action.

Design and Interpretation:
The design employs a clear left-to-right logic to link clinical efficacy with biological mechanisms:

Left Panel (Clinical Evidence): Summarizes the core outcomes from the meta-analysis, demonstrating JLD's significant benefits on **renal function** (reduced Scr, BUN, UAER), **glucose-lipid metabolism** (lowered HbA1c, TG, TC), and **inflammatory status** (reduced TNF-α, IL-6, hs-CRP), alongside a comparable safety profile to conventional therapy.

Right Panel (Mechanistic Insights): Features a simplified schematic of the potential multi-target mechanisms of JLD, highlighting its concurrent modulation of key signaling pathways, including **AMPK/PGC-1α** (for metabolic regulation and mitochondrial function), **NF-κB** (for anti-inflammation), and **Nrf2** (for antioxidative stress and renal protection).

Central Connection: Arrows and labels explicitly link the clinical improvements on the left to their corresponding mechanistic pathways on the right, illustrating a direct **clinical-molecular correlation**.

Central Conclusion:
The graphical abstract concludes that Jinlida Granule is a safe and effective adjuvant therapy for DKD, providing multi-dimensional benefits through its multi-target mechanisms, and representing a promising integrative medicine approach.

4 Supplementary Material S4. Detailed Risk of Bias Assessment

Assessment of evidence quality for each outcome

Quality assessment							No of patients		Effect		Quality	Importance
No of studies	Design	Risk of bias	Inconsistency	Indirectness	Imprecision	Other considerations	Treatment	Control	Relative (95% CI)	Absolute		
**Serum Creatinine (Scr) (Better indicated by lower values)**												
8	randomised trials	serious[1]	serious[2]	no serious indirectness	no serious imprecision	none	346	345	–	MD 30.54 μmol/L lower (37.61 to 23.47 lower)	LOW	CRITICAL
**Blood Urea Nitrogen (BUN) (Better indicated by lower values)**												
9	randomised trials	serious[1]	serious[2]	no serious indirectness	no serious imprecision	none	342	342	–	MD 0.95 mmol/L lower (1.29 to 0.60 lower)	LOW	CRITICAL
**Urinary Albumin Excretion Rate (UAER) (Better indicated by lower values)**												
7	randomised trials	serious[1]	serious[2]	no serious indirectness	no serious imprecision	none	301	301	–	MD 27.09 mg/24h lower (29.16 to 25.02 lower)	LOW	CRITICAL

Quality assessment							No of patients		Effect		Quality	Importance
No of studies	Design	Risk of bias	Inconsistency	Indirectness	Imprecision	Other considerations	Treatment	Control	Relative (95% CI)	Absolute		
**24-hour Urinary Protein (24hUP) (Better indicated by lower values)**												
4	randomised trials	serious[1]	serious[2]	no serious indirectness	serious[3]	none	112	113	–	MD 1.06 g/24h lower (1.27 to 0.84 lower)	VERY LOW	CRITICAL
**Fasting Blood Glucose (FBG) (Better indicated by lower values)**												
5	randomised trials	serious[1]	serious[2]	no serious indirectness	no serious imprecision	none	239	238	–	MD 0.83 mmol/L lower (1.37 to 0.30 lower)	LOW	IMPORTANT
**2-hour Postprandial Blood Glucose (2hPG) (Better indicated by lower values)**												
6	randomised trials	serious[1]	serious[2]	no serious indirectness	no serious imprecision	none	239	238	–	MD 1.25 mmol/L lower (2.13 to 0.37 lower)	LOW	IMPORTANT
**Glycated Hemoglobin (HbA1c) (Better indicated by lower values)**												
5	randomised trials	serious[1]	serious[2]	no serious indirectness	no serious imprecision	none	239	238	–	MD 0.86% lower (1.23 to 0.49 lower)	LOW	IMPORTANT

Quality assessment							No of patients		Effect		Quality	Importance
No of studies	Design	Risk of bias	Inconsistency	Indirectness	Imprecision	Other considerations	Treatment	Control	Relative (95% CI)	Absolute		
**HOMA-IR (Better indicated by lower values)**												
2	randomised trials	serious[1]	no serious inconsistency	no serious indirectness	serious[3]	none	107	107	–	MD 0.42 lower (0.54 to 0.31 lower)	VERY LOW	IMPORTANT
**Triglycerides (TG) (Better indicated by lower values)**												
3	randomised trials	serious[1]	serious[2]	no serious indirectness	serious[3]	none	150	149	–	MD 1.18 mmol/L lower (1.78 to 0.59 lower)	VERY LOW	IMPORTANT
**Total Cholesterol (TC) (Better indicated by lower values)**												
3	randomised trials	serious[1]	serious[2]	no serious indirectness	serious[3]	none	150	149	–	MD 0.99 mmol/L lower (1.88 to 0.10 lower)	VERY LOW	IMPORTANT
**hs-CRP (Better indicated by lower values)**												
3	randomised trials	serious[1]	serious[2]	no serious indirectness	serious[3]	none	142	142	–	MD 2.53 mg/L lower (3.13 to 1.93 lower)	VERY LOW	IMPORTANT

Quality assessment							No of patients		Effect		Quality	Importance
No of studies	Design	Risk of bias	Inconsistency	Indirectness	Imprecision	Other considerations	Treatment	Control	Relative (95% CI)	Absolute		
**TNF-α (Better indicated by lower values)**												
4	randomised trials	serious[1]	serious[2]	no serious indirectness	serious[3]	none	193	193	–	MD 4.36 ng/L lower (6.58 to 2.14 lower)	VERY LOW	IMPORTANT
**IL-6 (Better indicated by lower values)**												
3	randomised trials	serious[1]	serious[2]	no serious indirectness	serious[3]	none	142	142	–	MD 14.82 ng/L lower (26.52 to 3.11 lower)	VERY LOW	IMPORTANT
**VEGF (Better indicated by lower values)**												
5	randomised trials	serious[1]	serious[2]	no serious indirectness	no serious imprecision	none	252	252	–	MD 26.87 pg/mL lower (39.17 to 14.56 lower)	LOW	IMPORTANT
**IGF-1 (Better indicated by lower values)**												
3	randomised trials	serious[1]	serious[2]	no serious indirectness	serious[3]	none	175	175	–	MD 22.86 ng/mL lower (36.02 to 9.70 lower)	VERY LOW	IMPORTANT

Quality assessment							No of patients		Effect		Quality	Importance
No of studies	Design	Risk of bias	Inconsistency	Indirectness	Imprecision	Other considerations	Treatment	Control	Relative (95% CI)	Absolute		
**Overall Effective Rate (Better indicated by higher values)**												
7	randomised trials	serious[1]	no serious inconsistency	no serious indirectness	no serious imprecision	none	412	412	RR 1.18 (1.12 to 1.23)	144 more per 1000 (from 96 more to 184 more)	LOW	IMPORTANT
**Adverse Event Rate (Better indicated by lower values)**												
9	randomised trials	serious[1]	no serious inconsistency	no serious indirectness	serious[3]	**none**	154	153	RR 0.86 (0.50 to 1.48)	46 fewer per 1000 (from 72 fewer to 18 more)	VERY LOW	IMPORTANT

[1] All included studies were judged as having "some concerns" on the Cochrane RoB 2 tool, primarily due to absence of blinding of participants, healthcare providers, and outcome assessors (Domains 2 and 4), and lack of prospective trial registration (Domain 5).

[2] Large differences in the effect size of each study point or small overlap of confidence intervals, or large heterogeneity.

[3] Small sample size or wide confidence interval.

Assessment of risk of bias for each included study using the Cochrane Risk of Bias tool

地骨皮

5 Supplementary Material S5. Botanical Information of Jinlida Granule Composition

5.1 Part 1: Botanical Information of Jinlida Granule Composition

**Chinese Name**	**Latin Name (Full with Authority)**	**Plant Family**	**Part Used**	**Weight Ratio (%)**
黄精	*Polygonatum kingianum* Collett & Hemsl.	Asparagaceae	Rhizome	8.60
麦冬	*Ophiopogon japonicus* (Thunb.) Ker Gawl.	Asparagaceae	Tuber	8.60
山茱萸	*Cornus officinalis* Siebold & Zucc.	Cornaceae	Fruit	8.60
葛根	*Pueraria montana* var. *lobata* (Willd.) Maesen & S.M.Almeida ex Sanjappa & Predeep	Fabaceae	Root	8.60
荔枝核	*Litchi chinensis* Sonn.	Sapindaceae	Seed	8.60
人参	*Panax ginseng* C.A.Mey.	Araliaceae	Root and Rhizome	6.49
地黄	*Rehmannia glutinosa* (Gaertn.) Libosch. ex Fisch. & C.A.Mey.	Orobanchaceae	Root	6.49
丹参	*Salvia miltiorrhiza* Bunge	Lamiaceae	Root and Rhizome	5.63
何首乌	*Reynoutria multiflora* (Thunb.) Moldenke	Polygonaceae	Prepared Root	5.24
茯苓	*Wolfiporia cocos* (F.A. Wolf) Ryvarden & Gilb.	Polyporaceae	Sclerotium	5.24
地骨皮	*Lycium chinense* Mill.	Solanaceae	Root Bark	5.24

Chinese Name	Latin Name (Full with Authority)	Plant Family	Part Used	Weight Ratio (%)
苍术	*Atractylodes lancea* (Thunb.) DC.	Asteraceae	Rhizome (Stir-baked)	4.30
知母	*Anemarrhena asphodeloides* Bunge	Asparagaceae	Rhizome	4.30
苦参	*Sophora flavescens* Aiton	Fabaceae	Root	3.52
佩兰	*Eupatorium fortunei* Turcz.	Asteraceae	Aerial Part	3.52
黄连	*Coptis chinensis* Franch.	Ranunculaceae	Rhizome	3.52
淫羊藿	*Epimedium brevicornu* Maxim.	Berberidaceae	Leaf (Processed)	3.52

Botanical composition of Jinlida Granule. All plant names and families were verified via the Medicinal Plant Names Services (MPNS) portal (http://mpns.kew.org/mpns-portal/), accessed on 2025-11-19. As this study is a meta-analysis of previously conducted clinical trials, the original voucher specimen numbers for the specific plant materials used in the commercial batches of Jinlida Granule were not available. The weight ratios are based on the disclosed formula composition.

5.2 Part 2: Chemical Analysis Evidence for Jinlida Granule Quality Control

Component (Chinese Materia Medica)	Type of Chemical Analysis Evidence	Data Source (Literature/Standard/Database)	Key Findings/Explanation

Component (Chinese Materia Medica)	Type of Chemical Analysis Evidence	Data Source (Literature/Standard/Database)	Key Findings/Explanation
**Jinlida Granule (Overall)**	Thin Layer Chromatography (TLC) Identification	China Modern Applied Pharmacy, 2014, 31(2): 213-217	Established TLC identification methods for ginseng, salvia, pueraria, coptis, anemarrhena, prepared fleeceflower root and epimedium using **four separate TLC plates**, not simultaneous identification on a single plate.
**Sophora (苦参)**	Content Determination (HPLC)	China Modern Applied Pharmacy, 2014, 31(2): 213-217	HPLC method for matrine determination using aminopropyl-bonded silica gel column with acetonitrile-absolute ethanol-2% phosphoric acid solution (84:7:9) as mobile phase. Good linear relationship (r=0.9999) with average recovery of 100.4%.
**Sophora (苦参)**	Content Determination (UPLC)	China Modern Drug Application, 2018, 12(20): 216-218	UPLC method for matrine determination using Thermo aminopropyl column with acetonitrile-absolute ethanol-2% phosphoric acid solution (88:6:6), flow rate 0.2ml/min. Good linear relationship (r=0.9992) with average recovery of 99.6%.
**Sophora (苦参)**	Thin Layer Chromatography (TLC) Identification	China Modern Drug Application, 2018, 12(18): 217-218	Established TLC method for simultaneous identification of matrine, oxymatrine and sophoridine. The method is simple, reproducible and reliable.
**Ginseng, Salvia, Pueraria, Coptis, Anemarrhena, Prepared Fleeceflower Root, Epimedium**	Thin Layer Chromatography (TLC) Identification	China Modern Applied Pharmacy, 2014, 31(2): 213-217	These medicinal materials were included in the overall TLC identification study of Jinlida Granule and corresponding identification methods were established using **four separate TLC plates**.
**All 17 medicinal materials**	Legal Composition and Standard	Pharmacopoeia of the People's Republic of China (2015 Edition, Volume I)	The national drug standard for Jinlida Granule is included in the pharmacopoeia. Its composition includes: Ginseng, Polygonatum, Stir-fried Atractylodes, Sophora, Ophiopogon, Rehmannia, Prepared Fleeceflower Root, Cornus, Poria, Eupatorium, Coptis, Anemarrhena, Processed Epimedium, Salvia, Pueraria, Lychee Seed, and Lycium Root Bark.

These analytical methods provide verification of the identity and quality of the herbal materials used in Jinlida Granule, in accordance with journal requirements for situations where voucher specimens are unavailable.

Compliance Statement

This supplementary material addresses the requirements of the Journal of Ethnopharmacology regarding plant material identification as follows:

1. **Plant Names:** All plant species mentioned in the manuscript are cited with their full botanical names, including authorities, following the standards set by the Medicinal Plant Names Services (MPNS) portal.
2. **Voucher Specimens:** In accordance with journal policy, where voucher specimens have not been retained, comprehensive chemical profiles of the plant material have been provided.
3. **Traditional Use Context:** The activity studied relates to the traditional use of Jinlida Granule, with reference to its inclusion in the Pharmacopoeia of the People's Republic of China.

6 Supplementary Material S6. Subgroup analysis

6.1 Subgroup analysis for renal function indicators

6.1.1 Forest Plots for Subgroup Analysis of Renal Function Indicators

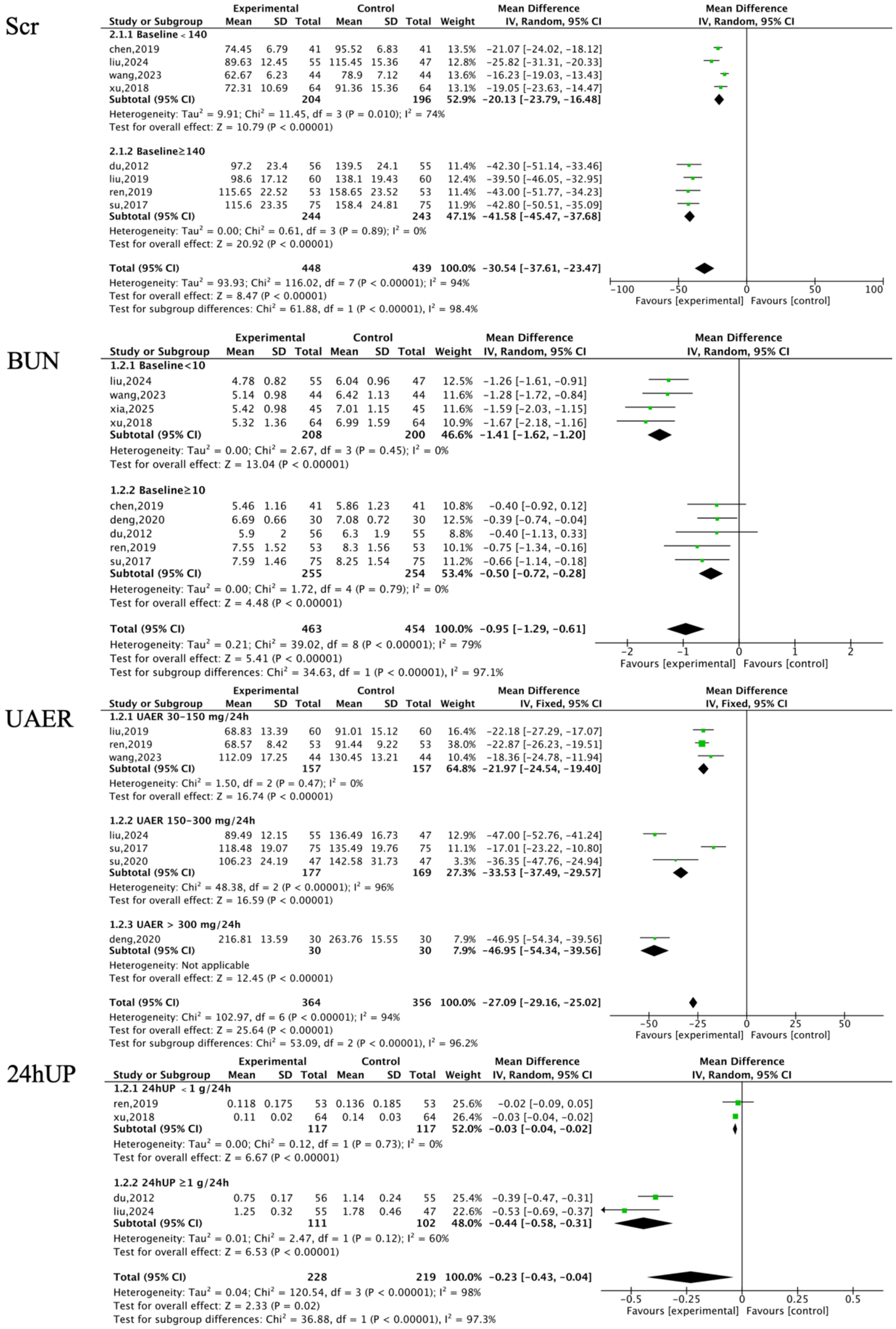

Forest plots illustrating subgroup analyses based on baseline renal function parameters

6.1.2 Table renal content：Subgroup analysis for Scr, BUN, UAER, and 24hUP

Indicator	Subgrouping Dimension	Subgroup Category	No. of comparisons	Result: MD (95% CI)	Heterogeneity			p-value for subgroup difference	Key Findings
					p-value for overall effect	p-value for heterogeneity	I^2 (%)		
Scr	-	All comparisons	8	-30.54 (-37.61, -23.47)	<0.001	<0.001	94.0	-	The analysis identified baseline Scr level as the principal factor explaining heterogeneity. A distinct pattern emerged: the treatment effect was proportional to the baseline level in patients with milder impairment, but became strong, consistent, and independent of the baseline level in patients with more severe renal dysfunction.
	Baseline Scr level	<140 μmol/L	4	-20.13 (-23.79, -16.48)	<0.001	0.010	73.8	<0.001	
	Baseline Scr level	≥140 μmol/L	4	-41.58 (-45.47, -37.68)	<0.001	0.894	0.0		
BUN	-	All comparisons	9	-0.95 (-1.29, -0.61)	<0.001	<0.001	79.5	-	Subgroup analysis successfully resolved the high heterogeneity, revealing that the treatment effect is closely tied to baseline renal function. Patients with better-preserved kidney function exhibited a more pronounced reduction in BUN, whereas those with more impaired function still showed a consistent, though smaller, benefit.
	Baseline BUN level	<10 mmol/L	4	-1.41 (-1.62, -1.20)	<0.001	0.446	0.0	<0.001	
	Baseline BUN level	≥10 mmol/L	5	-0.50 (-0.72, -0.28)	<0.001	0.787	0.0		
UAER	-	All comparisons	7	-29.87	<0.001	<0.001	94.2	-	The efficacy of the treatment demonstrated a general trend of being proportional to the baseline

Indicator	Subgrouping Dimension	Subgroup Category	No. of comparisons	Result: MD (95% CI)	Heterogeneity			p-value for subgroup [illegible]	Key Findings
				(-38.95, -20.78)					UAER level, suggesting that patients with higher baseline proteinuria derive greater benefit. Heterogeneity was low in early-stage patients but high in the clinical proteinuria group, partly attributable to differences in treatment duration.
	Baseline UAER level	30-150 mg/24h (early-stage)	3	-21.97 (-24.54, -19.40)	<0.001	0.473	0.0		
	Baseline UAER level	150-300 mg/24h (clinical proteinuria)	3	-42.86 (-53.04, -32.68)	<0.001	<0.001	95.9	<0.001	
	Baseline UAER level	>300 mg/24h (massive proteinuria)	1	-46.95 (-55.22, -38.68)	<0.001	-	-		
	-	All comparisons	4	-0.23 (-0.43, -0.04)	<0.001	<0.001	97.5	-	
24hUP	Baseline 24hUP level	<1g/24h	2	-0.03 (-0.04, -0.02)	<0.001	0.734	0.0		A clear biological gradient was observed, where the magnitude of proteinuria reduction was substantially greater in patients with high baseline levels. This indicates that the absolute treatment benefit is most significant in patients with advanced disease, while a "ceiling effect" may limit observable benefits in those with near-normal baseline levels.
	Baseline 24hUP level	≥1g/24h	2	-0.44 (-0.58, -0.31)	<0.001	0.116	59.6	<0.001	

Abbreviations: Scr: Serum creatinine; BUN: Blood urea nitrogen; UAER: Urinary albumin excretion rate; 24hUP: 24-hour urinary protein; MD: Mean difference; CI: Confidence interval.

Note: All analyses used random-effects models. Subgroup analyses were performed to explore sources of heterogeneity.

This table provides comprehensive subgroup analysis results exploring sources of heterogeneity across various outcome measures.

6.2 Subgroup analysis for glucose and lipid metabolism indicators

6.2.1 Forest Plots for Subgroup Analysis of Glucose and Lipid Metabolism Indicators

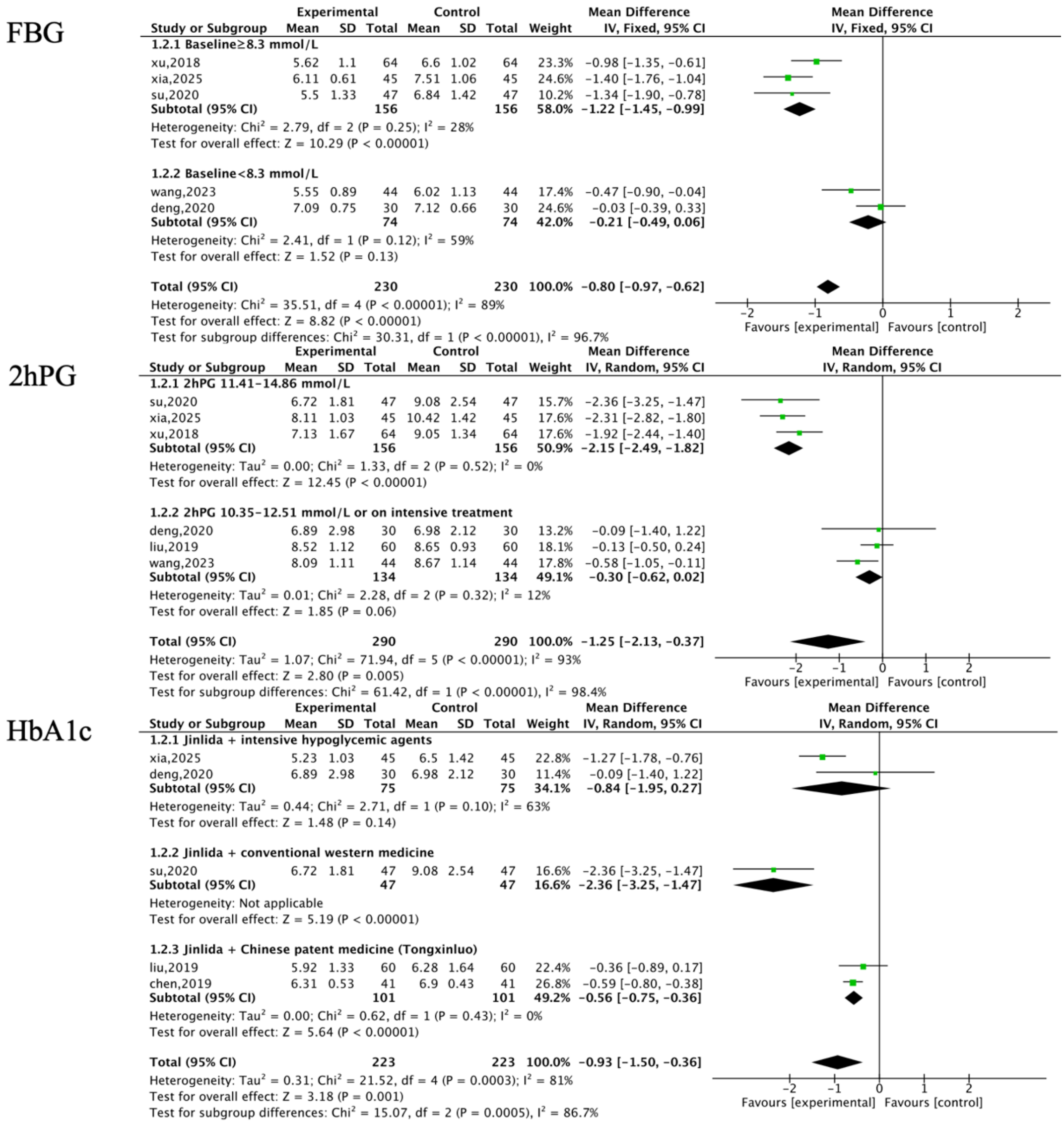

Forest plots illustrating subgroup analyses based on baseline glucose and lipid parameters and combination medication regimens.

6.2.2 Table glucose content: Subgroup analysis for FBG, 2hPG, HbA1c, TG, and TC

Indicator	Subgrouping	Subgroup	No. of	Result:	Heterogeneity	p-value	Key Findings

	Dimension	Category	comparisons	MD (95% CI)	p-value for overall effect	p-value for heterogeneity	I² (%)	for subgroup difference	
FBG	-	All comparisons	5	-0.835 (-1.371, -0.299)	<0.001	<0.001	88.7	-	Subgroup analysis revealed that the glucose-lowering efficacy is highly dependent on baseline glycemic status. A pronounced effect was observed in patients with poorly controlled hyperglycemia, whereas no significant additional benefit was found in patients whose glucose was already near target levels, indicating a clear ceiling effect.
	Baseline FBG level	≥8.3 mmol/L	3	-1.224 (-1.505, -0.944)	<0.001	0.248	28.4	<0.001	
	Baseline FBG level	<8.3 mmol/L	2	-0.234 (-0.664, 0.196)	0.128	0.120	58.5		
2hPG	-	All comparisons	6	-1.253 (-2.131, -0.374)	<0.001	<0.0001	93.0	-	Efficacy was distinctly stratified by the degree of baseline postprandial dysmetabolism. A robust and consistent glucose-lowering effect was confirmed in patients with significant baseline hyperglycemia, whereas the effect was minimal and uncertain in patients with relatively well-controlled glucose levels.
	Baseline metabolic disorder degree	High baseline disorder (2hPG 11.41-14.86 mmol/L)	3	-2.154 (-2.493, -1.815)	<0.001	0.515	0.0	<0.001	
	Baseline metabolic disorder degree	Relatively stable control (2hPG 10.35-12.51 mmol/L)	3	-0.299 (-0.616, 0.018)	0.044	0.320	12.1		
HbA1c	-	All comparisons	5	-0.847% (-1.224%,-0.470%)	<0.001	<0.001	86.0	-	The analysis successfully attributed heterogeneity to the

Indicator	Subgrouping Dimension	Subgroup Category	No. of comparisons	Result: MD	Heterogeneity			p-value for	Key Findings
	Combination medication regimen	Jinlida + intensive hypoglycemic agents	2	-1.116% (-1.465%,-0.767%)	<0.001	<0.001	67.3	<0.001	background glucose-lowering regimen. The most substantial HbA1c reduction was achieved when Jinlida was combined with potent glucose-lowering agents, suggesting synergistic effects, while a consistent but more modest effect was observed with conventional combination therapies.
	Combination medication regimen	Jinlida + conventional western medicine	1	-1.250% (-1.465%,-1.035%)	<0.001	-	-		
	Combination medication regimen	Jinlida + Chinese patent medicine (Tongxinluo)	2	-0.557% (-0.723%,-0.391%)	<0.001	0.339	0.0		
TG	-	All comparisons	3	-1.184 (-1.781, -0.587)	<0.001	<0.001	92.1	-	The analysis demonstrated a classic baseline-dependent treatment response. A significant lipid-lowering effect was exclusive to patients with baseline hypertriglyceridemia, whereas no meaningful effect was observed in patients with normal baseline levels, highlighting the critical role of patient selection.
	Baseline TG level	≥2.0 mmol/L	2	-1.584 (-1.922, -1.247)	<0.001	0.01	78.6	<0.001	
	Baseline TG level	<2.0 mmol/L	1	-0.070 (-0.158, 0.018)	0.11	-	-		
TC	-	All comparisons	3	-0.993 (-1.877, -0.110)	0.027	<0.001	92.9	-	Heterogeneity was primarily driven by the specific combination therapy. The analysis suggested a potential for enhanced cholesterol-lowering when Jinlida was paired with certain antihyperglycemic
	Combination medication regimen	Jinlida + Dapagliflozin	1	-1.039 (-2.803, 0.725)	0.24	-	-	<0.001	
	Combination medication	Jinlida + Benazepril	1	-0.553 (-1.327,	0.16	-	-		

Indicator	Subgrouping Dimension	Subgroup Category	No. of comparisons	Result: MD	Heterogeneity			p-value for	Key Findings
	regimen			0.221)					drugs, whereas the effect varied significantly with other combination regimens.
	Combination medication regimen	Jinlida + Chinese patent medicine (Tongxinluo)	1	-1.419 (-2.408, -0.431)	0.005	-	-		

Abbreviations：FBG: Fasting blood glucose; 2hPG: 2-hour postprandial glucose; HbA1c: Glycated hemoglobin; TG: Triglycerides; TC: Total cholesterol; MD: Mean difference; CI: Confidence interval.

Note: All analyses used random-effects models. Subgroup analyses were performed to explore sources of heterogeneity.

6.3 Subgroup Analysis for Inflammatory Markers

6.3.1 Forest Plots for Subgroup Analysis of Inflammatory Markers

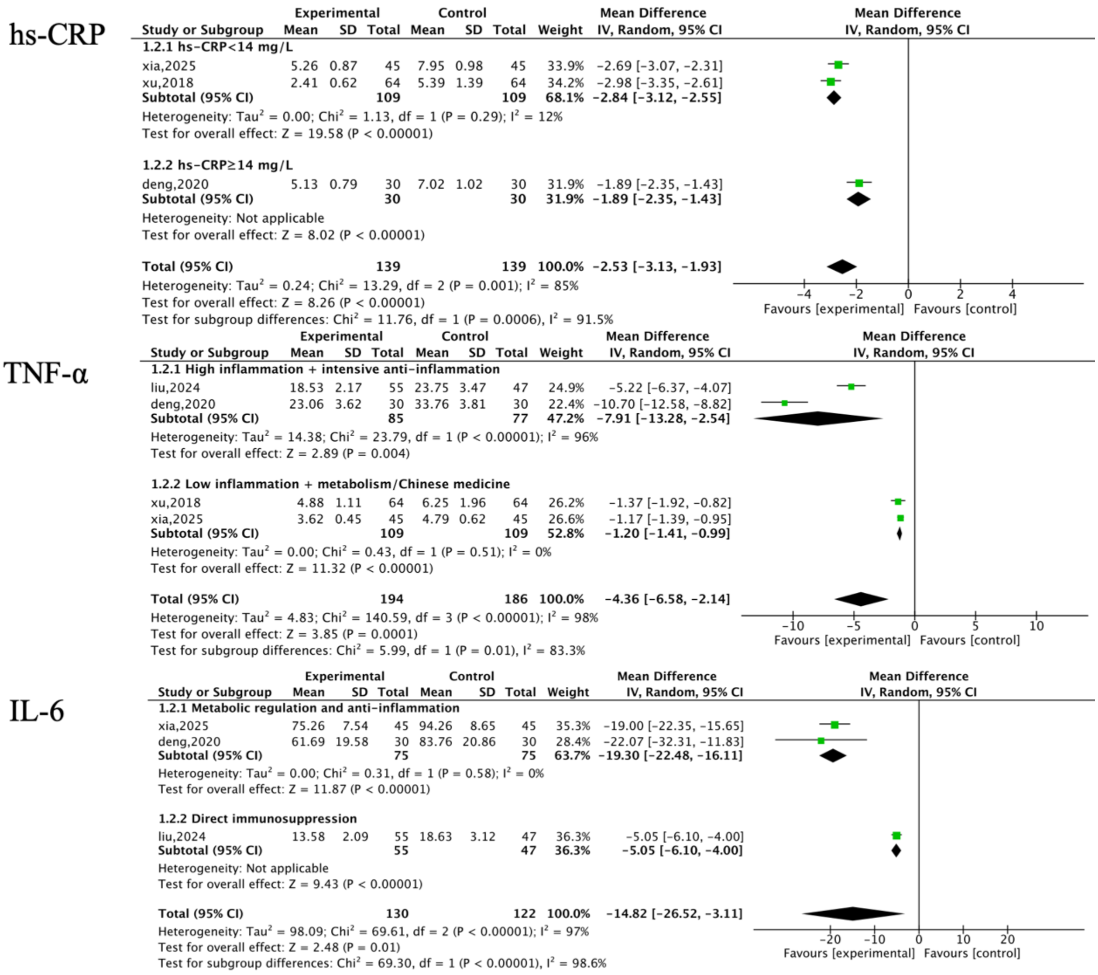

Forest plots illustrating subgroup analyses based on baseline inflammatory levels and combination mechanisms.

6.3.2 Table inflammatory content: Subgroup analysis for hs-CRP, TNF-α, and IL-6

Indicator	Subgrouping Dimension	Subgroup Category	No. of comparisons	Result: MD (95% CI)	Heterogeneity			p-value for subgroup difference	Key Findings
					p-value for overall effect	p-value for heterogeneity	I² (%)		
hs-CRP	-	All comparisons	3	-2.534<br>(-3.135, -1.932)	<0.001	<0.01	84.9	-	Subgroup analysis revealed that the anti-inflammatory efficacy is strongly dependent on baseline inflammatory status. A consistent and pronounced reduction was observed in patients with low baseline inflammation, whereas the effect was attenuated in patients with high baseline levels, suggesting a potential ceiling effect in advanced inflammatory states.
	Baseline level	<14 mg/L	2	-2.838<br>(-3.123, -2.554)	<0.001	0.28	11.6	<0.001	
	Baseline level	≥14 mg/L	1	-2.132<br>(-2.500, -1.764)	<0.001	-	-		
TNF-α	-	All comparisons	4	-4.361<br>(-6.585, -2.138)	0.0002	<0.001	97.9	-	A compound subgrouping strategy (baseline level + combination mechanism) successfully deconstructed the heterogeneity. Efficacy was most substantial in patients with high baseline inflammation combined with potent anti-inflammatory agents, yet with significant individual variability. In contrast, a stable and consistent effect was achieved in patients with low baseline inflammation using metabolic or traditional Chinese medicine regimens.
	Composite grouping	High inflammation + intensive anti-inflammation	2	-7.907<br>(-13.276,-2.538)	0.004	<0.01	95.8	0.01	
	Composite grouping	Low inflammation + metabolism/Chinese medicine	2	-1.198<br>(-1.406, -0.991)	<0.001	0.89	0.0		
IL-6	-	All comparisons	3	-14.816<br>(-26.521, -3.112)	0.013	<0.001	97.1	-	Heterogeneity was primarily attributed to the mechanism of the background anti-inflammatory regimen. A stable and significant reduction in IL-6 was specifically observed when Jinlida was combined with metabolic-regulating anti-inflammatory drugs (e.g., SGLT2i, GLP-1 RA), suggesting a synergistic effect within this pathway, whereas the effect was less pronounced when combined with direct immunosuppressants.
	Combination mechanism	Metabolic regulation and anti-inflammation	2	-19.297<br>(-22.484,-16.111)	<0.001	0.98	0.0	<0.001	
	Combination mechanism	Direct immunosuppression	1	-5.050<br>(-6.100, -4.000)	<0.001	-	-		

Indicator	Subgrouping Dimension	Subgroup Category	No. of comparisons	Result: MD (95% CI)	Heterogeneity	p-value for subgroup difference	Key Findings

Abbreviations: hs-CRP: High-sensitivity C-reactive protein; TNF-α: Tumor necrosis factor-α; IL-6: Interleukin-6; MD: Mean difference; CI: Confidence interval.
Note: All analyses used random-effects models. Subgroup analyses were performed to explore sources of heterogeneity.

6.4 Subgroup Analysis for Growth Factors

6.4.1 Forest Plots for Subgroup Analysis of Growth Factors

VEGF

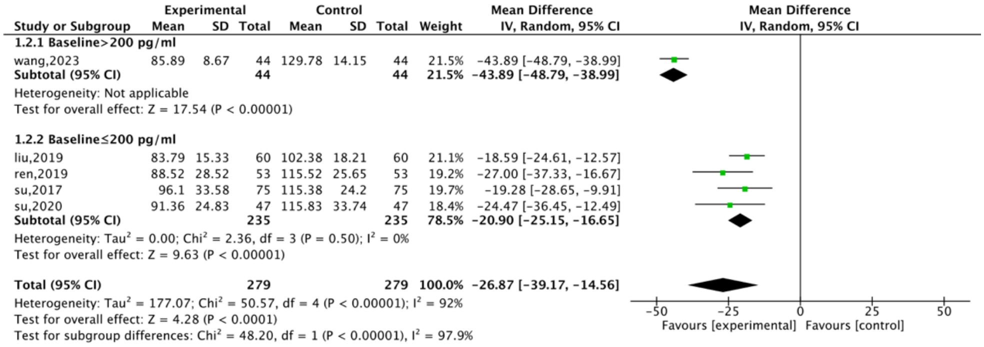

IGF-1

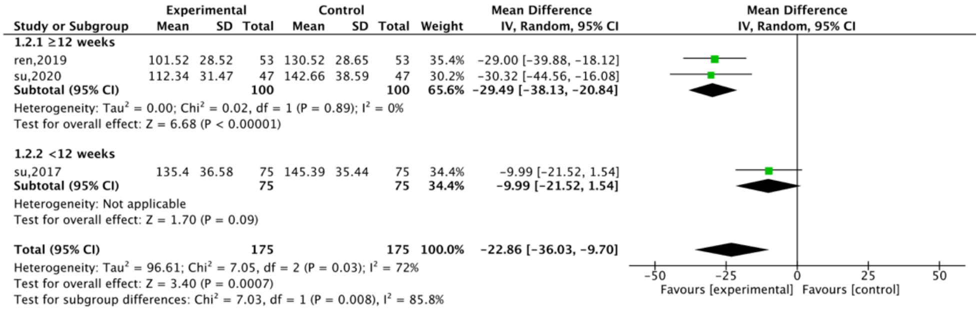

Forest plots illustrating subgroup analyses based on baseline levels and treatment duration.

6.4.2 Table growth content: Subgroup analysis for VEGF and IGF-1

Indicator	Subgrouping Dimension	Subgroup Category	No. of comparisons	Result: MD (95% CI)	Heterogeneity			p-value for subgroup difference	Key Findings
					p-value for overall effect	p-value for heterogeneity	I^2 (%)		
VEGF	-	All comparisons	5	-26.867 (-39.173, -14.560)	<0.001	<0.001	92.1	-	Subgroup analysis identified baseline VEGF level as the primary source of heterogeneity. A consistent and significant reduction was observed in the typical patient population with low-to-moderate baseline levels, whereas the effect was exaggerated in a single study with exceptionally high baseline levels, highlighting the critical role of baseline stratification for accurate efficacy estimation.
	Baseline level	>200 pg/ml	1	-43.890 (-48.790, -38.990)	<0.001	-	-	<0.001	
	Baseline level	≤200 pg/ml	4	-20.899 (-25.151, -16.647)	<0.001	0.501	0.0		
IGF-1	-	All comparisons	3	-22.86 (-36.02, -9.70)	<0.001	0.03	71.6	-	The analysis revealed that treatment duration was a key modifier of the effect. A significant and highly consistent reduction in IGF-1 was specifically demonstrated in the subgroup with a standard treatment duration (≥12 weeks), whereas the effect was diminished in a shorter-duration study, underscoring the importance of adequate intervention time for a stable treatment response.
	Treatment duration	≥12 weeks	2	-29.66 (-40.58, -18.74)	<0.001	0.76	0.0	0.008	
	Treatment duration	<12 weeks	1	-9.99 (-21.52, 1.54)	<0.001	-	-		

Abbreviations: VEGF: Vascular endothelial growth factor; IGF-1: Insulin-like growth factor-1; MD: Mean difference; CI: Confidence interval.
Note: All analyses used random-effects models. Subgroup analyses were performed to explore sources of heterogeneity.

7 Supplementary Material S7. Sensitivity analysis

7.1 Supplementary Figure : Sensitivity analysis

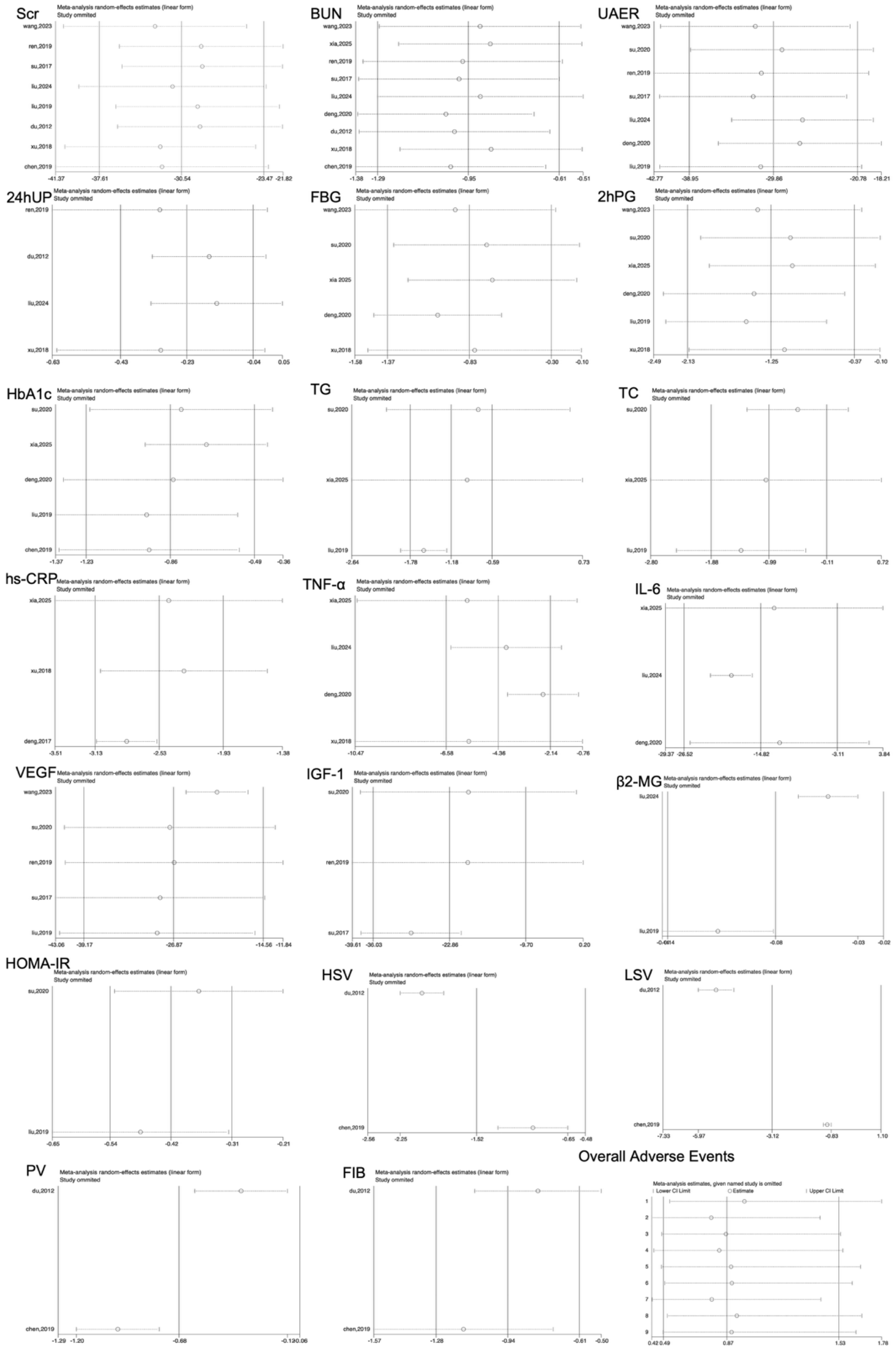

7.2 Supplementary Table : Sensitivity Analysis Results

Outcome	No. of Studies	Sensitivity Analysis Method	Key Findings	Robustness Conclusion
Serum Creatinine (Scr)	8	Leave-one-out analysis	After excluding any single study, the combined MD remained negative with 95% CI not including 0. The conclusion direction remained consistent.	Robust
Blood Urea Nitrogen (BUN)	9	Leave-one-out analysis	After excluding any single study, the combined MD remained negative with 95% CI not including 0. The conclusion direction remained consistent.	Robust
Urinary Albumin Excretion Rate (UAER)	7	Leave-one-out analysis	After excluding any single study, the combined MD remained negative with 95% CI not including 0. The conclusion direction remained consistent.	Robust
24-hour Urinary Protein (24hUP)	4	Leave-one-out analysis	After excluding any single study, the combined MD remained negative with 95% CI not including 0. The conclusion direction remained consistent.	Robust
Fasting Blood Glucose (FBG)	5	Leave-one-out analysis, Subgroup-based reanalysis, Combination regimen reanalysis, Methodological quality reanalysis	After excluding any single study, the combined MD remained negative with 95% CI not including 0. The effect was most stable in the high baseline subgroup (≥8.3 mmol/L).	Robust
2-hour Postprandial Glucose (2hPG)	6	Leave-one-out analysis	After excluding any single study, the combined MD remained negative with 95% CI not including 0. The conclusion direction remained consistent.	Robust
Glycated Hemoglobin (HbA1c)	5	Leave-one-out analysis	After excluding any single study, the combined MD remained negative with 95% CI not including 0. The conclusion direction remained consistent.	Robust
Triglycerides (TG)	3	Leave-one-out analysis	After excluding any single study, the combined MD remained negative with 95% CI not including 0. The conclusion direction remained consistent.	Robust
Total Cholesterol (TC)	3	Leave-one-out analysis	After excluding any single study, the combined MD remained negative with 95% CI not	Robust

Outcome	No. of Studies	Sensitivity Analysis Method	Key Findings	Robustness Conclusion
			including 0. The effect was most pronounced when combined with dapagliflozin.	
High-sensitivity C-reactive Protein (hs-CRP)	3	Leave-one-out analysis	After excluding any single study, the combined MD remained negative with 95% CI not including 0. The conclusion direction remained consistent.	Robust
Tumor Necrosis Factor-α (TNF-α)	4	Leave-one-out analysis	After excluding any single study, the combined MD remained negative with 95% CI not including 0. The conclusion direction remained consistent, though effect size varied.	Robust
Interleukin-6 (IL-6)	3	Leave-one-out analysis	After excluding any single study, the combined MD remained negative with 95% CI not including 0. The conclusion direction remained consistent.	Robust
Vascular Endothelial Growth Factor (VEGF)	5	Leave-one-out analysis	After excluding Wang 2023 (high baseline outlier), heterogeneity decreased from 92.1% to 0.0%, with MD changing from -26.867 to -20.899 pg/ml (still significant).	Robust after excluding outlier
Insulin-like Growth Factor-1 (IGF-1)	3	Leave-one-out analysis	After excluding any single study, the combined MD remained negative with 95% CI not including 0. The conclusion direction remained consistent.	Robust
β2-microglobulin (β2-MG)	2	Leave-one-out analysis	Extremely high heterogeneity ($I^2 = 91.5\%$) due to differences in patient severity and combination regimens. Limited interpretability of pooled results.	Cautious interpretation required
Homeostasis Model Assessment of Insulin Resistance (HOMA-IR)	2	Leave-one-out analysis, Model comparison	After excluding either study, the remaining study showed significant effect. Fixed and random effects models yielded consistent results.	Robust
High-cut viscosity	2	Leave-one-out analysis	Significant reduction observed but with high heterogeneity ($I^2 = 96.4\%$). Effect size unstable across sensitivity analyses.	Cautious interpretation required

Outcome	No. of Studies	Sensitivity Analysis Method	Key Findings	Robustness Conclusion
Low-cut viscosity	2	Leave-one-out analysis	Significant reduction observed but with extremely high heterogeneity ($I^2 = 99.3\%$). Wide confidence intervals indicate high uncertainty.	Cautious interpretation required
Plasma viscosity	2	Leave-one-out analysis	Significant reduction observed but with high heterogeneity ($I^2 = 93.4\%$). Effect size moderately stable.	Cautious interpretation required
Fibrinogen	2	Leave-one-out analysis	Significant reduction observed with moderate heterogeneity ($I^2 = 43.4\%$). Most reliable evidence among hemorheology indicators.	Robust
Overall Adverse Events	9	Leave-one-out analysis	After excluding any single study, the combined RR fluctuated between 0.787-0.972, with 95% CI all crossing 1.0, and I^2 always 0.0%.	Robust

This table provides detailed sensitivity analysis results assessing the robustness of the meta-analysis findings

8 Supplementary Material S8.Traditional Use and Ethnopharmacological Relevance Documentation

8.1 Traditional Chinese Medicine Theoretical Foundation

Traditional Chinese Medicine Theoretical Foundation		
Item	Content	Supporting References
TCM Disease Classification	Diabetic Kidney Disease is classified under "Xiaoke Nephropathy" in TCM theory	Introduction section of main text; Li et al., Am J Chin Med, 2025 (Ref 15); Chinese Guidelines for DKD Diagnosis and Treatment
Core	Qi and Yin deficiency progressing to spleen and	Introduction section of

Pathogenesis	kidney deficiency, with pathological products (blood stasis, phlegm, turbid toxins) obstructing the kidney collaterals, forming the key pathogenesis of "toxin damaging the kidney collaterals"	main text; Li et al., Am J Chin Med, 2025 (Ref 15); Deng et al., J Ethnopharmacol, 2024 (Ref 19)
Therapeutic Principle	"Treating from the Spleen" theory - fortifying the spleen to transport and transform nutrients, thereby regulating glucose metabolism and preventing complications	Introduction section of main text; Jinlida Granule approval documentation; TCM theoretical foundations

8.2 Jinlida Granule Formula Principles and Composition Rationale

**Jinlida Granule Formula Principles and Composition Rationale**		
**Item**	**Content**	**Supporting References**
Formula Strategy	Monarch-Minister-Assistant-Guide composition following strict TCM formulation principles to replenish Qi, nourish Yin, fortify the spleen, and activate collaterals	Table 1 in main text; TCM formulation theory
Key Herbs and Traditional Functions	• **Ginseng Radix et Rhizoma (Renshen)**: Greatly tonifies original Qi, strengthens spleen and lung<br>• **Polygonati Rhizoma (Huangjing)**: Nourishes Yin, tonifies spleen Qi<br>• **Salviae Miltiorrhizae Radix (Danshen)**: Activates blood, resolves stasis, clears collaterals<br>• **Puerariae Thomsonii Radix (Gegen)**: Generates fluids, alleviates thirst<br>• **Ophiopogonis Radix (Maidong)**: Nourishes Yin, promotes fluid production<br>• **Litchi Semen (Lizhihe)**: Regulates Qi, disperses stagnation, dissipates cold and relieves pain	Chinese Pharmacopoeia (2020 Edition); Traditional Chinese Materia Medica references; Multiple herbal mechanism studies (e.g., Liu et al., Front Endocrinol, 2023) provide modern pharmacological validation for these traditional functions

	• **Rehmanniae Radix (Dihuang)**: Clears heat, cools blood, nourishes Yin and promotes fluid production	

8.3 Modern Scientific Evidence Supporting Traditional Use

**Modern Scientific Evidence Supporting Traditional Use**		
**Item**	**Content**	**Supporting References**
Multi-target Mechanisms	• **Improves mitochondrial function via AMPK/PGC-1α pathway** (podocyte protection)<br>• **Exerts antioxidant effects via Nrf2 pathway activation**<br>• **Confers anti-inflammatory effects via NF-κB pathway inhibition**<br>• **Inhibits renal fibrosis via TGF-β1/Smad signaling pathway**<br>• **Attenuates renal injury via autophagy regulation**<br>• **Exerts renal protection via SIRT1/NF-κB pathway**	**Sun et al., Int J Mol Med, 2025 (Ref 17)** - AMPK/PGC-1α<br>**Tanase et al., Biomolecules, 2022 (Ref 18)**- Nrf2/antioxidant<br>**Deng et al., J Ethnopharmacol, 2024 (Ref 19)** - NF-κB/anti-inflammatory<br>**Chen et al., China J Tradit Chin Med Pharm, 2019 (Ref 35)** - TGF-β1/Smad/anti-fibrotic<br>**Liu et al., Front Endocrinol, 2023 (Ref 42)**- autophagy regulation<br>**Lu et al., Chinese Journal of Diabetes, 2016** - SIRT1/NF-κB pathway
Clinical Efficacy Evidence	• Significantly improves renal function indicators (serum creatinine, blood urea nitrogen, urinary protein excretion rate)<br>• Improves glucose and lipid metabolism (fasting blood glucose, HbA1c, triglycerides, total cholesterol)<br>• Reduces inflammatory markers (hs-CRP, TNF-α, IL-6)<br>• Demonstrates synergistic effects when combined with SGLT2 inhibitors, GLP-1 receptor agonists, etc.	Meta-analysis results in main text; **Xia et al., Acta Medicinae Sinica, 2025** - JLD + Dapagliflozin;<br>**Deng et al., Journal of Bethune Medical Science, 2020** - JLD + Liraglutide
Clinical Guideline	**Official Guideline Recommendations**<br>**Chinese Guidelines for Type 2 Diabetes**	Chinese Diabetes Society "Chinese Guidelines for Type 2

Recognition	**Prevention and Treatment Series**:<br>• 2017 Edition: First to add "Diabetes and Traditional Chinese Medicine" chapter, recommending Jinlida Granule as the only Chinese patent medicine for type 2 diabetes treatment<br>• 2020 Edition: Continued recommendation of Jinlida Granule for type 2 diabetes treatment<br>• 2024 Edition: Breakthrough recommendation for prediabetes treatment, reducing type 2 diabetes risk by 41%<br><br>**Other Official Guidelines and Consensus Recommendations**:<br>• International Traditional Chinese Medicine Diabetes Diagnosis and Treatment Guidelines<br>• Evidence-based Clinical Practice Guidelines for Diabetes in Traditional Chinese Medicine<br>• 2023 Integrated Traditional Chinese and Western Medicine Guidelines for Type 2 Diabetes (first-line recommendation)	Diabetes Prevention and Treatment" (2017/2020/2024 Editions);<br>"International Traditional Chinese Medicine Diabetes Diagnosis and Treatment Guidelines";<br>"2023 Integrated Traditional Chinese and Western Medicine Guidelines for Type 2 Diabetes"
Regulatory Status	**Official Approval Information**<br>• **Approval Year**: 2005<br>• **Approval Number**: National Medicine Approval Number Z20050845<br>• **Manufacturer**: Shijiazhuang Yiling Pharmaceutical Co., Ltd.<br>• **National Drug Standard**: YBZ30982005-2010Z<br>• **Medical Insurance**: National Medical Insurance Category B<br>• **Essential Drug List**: National Essential Drugs List	National Medical Products Administration Approval Documents (Approval Number: Z20050845);<br>"National Basic Medical Insurance, Work Injury Insurance, and Maternity Insurance Drug List";<br>"National Essential Drugs List"

8.4 Integration with Contemporary DKD Pathophysiology

Integration with Contemporary DKD Pathophysiology		
**Item**	**Content**	**Supporting References**

Alignment with Modern Pathophysiology	Jinlida's multi-target effects address key DKD pathways: metabolic memory, mitochondrial dysfunction, chronic inflammation, and fibrosis - providing scientific basis for its traditional "multi-target" approach	**DeFronzo et al., Nat Rev Nephrol, 2021 (Ref 3)** - DKD multi-mechanism pathophysiology<br>**Ansari et al., Cytokine Growth Factor Rev, 2025 (Ref 4)** - core role of inflammation and fibrosis<br>**Mitrofanova et al., Cells, 2022 (Ref 6)** - mitochondrial-inflammation connection
Bridging Traditional and Modern Medicine	The formula's holistic approach aligns with contemporary understanding of DKD as a complex, multi-mechanism disease requiring comprehensive intervention beyond single-target therapies	**Wanner et al., Adv Ther, 2025 (Ref 11)** - call for multi-drug GDMT combination<br>**Discussion section of main text** - systematic discussion of JLD's multi-target characteristics aligning with modern GDMT concept
Addressing Guideline-Practice Gap	Provides an effective integrated Chinese-Western medicine complementary strategy for the clinical challenge of underutilization of guideline-recommended drugs like SGLT2i in real-world practice	**Forbes et al., EClinicalMedicine, 2024 (Ref 12)** - real-world application gap of SGLT2i<br>**Discussion section of main text** - Jinlida as potential strategy to bridge guideline-practice gap

This table provides comprehensive documentation of the traditional use and ethnopharmacological relevance of Jinlida Granule, aligning with Journal of Ethnopharmacology requirements.

Summary of Ethnopharmacological Relevance

Jinlida Granule represents a clinically implemented embodiment of TCM theory for diabetes and its complications, specifically developed based on the "Treating from the Spleen" principle for glucose metabolism disorders. Its traditional use for "Xiaoke" (wasting-thirst) conditions is robustly validated by modern science, which demonstrates its multi-target mechanisms in addressing core DKD pathophysiology.

9 Supplementary Material S10. Statistical Analysis Code - STATA Commands

Note: This document provides the complete STATA code used for all statistical analyses in the systematic review and meta-analysis. The code is organized by variable type and analysis objective for clarity and reproducibility.

Continuous Variables Meta-Analysis

Analysis Objective	STATA Command	Description
Primary Meta-Analysis	metan n_exp mean_exp sd_exp n_cotr mean_cotr sd_cotr, fixed label(namevar=study) nostandard	Fixed-effects meta-analysis of continuous outcomes using raw means and SDs
Random-Effects Model	metaan _ES _seES, label(study) dl forest	DerSimonian-Laird random-effects model with forest plot
Meta-Regression	metareg _ES, wsse(_seES) eform	Meta-regression exploring heterogeneity (exponentiated coefficients)
Influence Analysis	metainf _ES _seES, id(study) random forest	Influence analysis showing effect of omitting each study
Funnel Plot	metafunnel _ES _seES	Funnel plot for visual assessment of publication bias
Egger's Test	metabias6 _ES _seES	Statistical test for funnel plot asymmetry
Trim and Fill Analysis	metatrim _ES _seES, eform funnel	Trim and fill method to adjust for publication bias

Efficacy Index Variables (Ordinal Outcomes)

Analysis Objective	STATA Command	Description
Data Reshaping	reshape long case, i(i study) j(effact)	Reshape data from wide to long format for ordinal analysis
Ordinal Logistic Regression	by study, sort: ologit effact treatment treatment [fweight= case]	Study-level ordinal logistic regression
Meta-Analysis of Log Odds	metan lnor selnor, fixed eform label(namevar= study)	Fixed-effects meta-analysis of log odds ratios
Random-Effects Model	meta lnor selnor	Random-effects meta-analysis of log odds ratios
Influence Analysis	metainf lnor selnor, id(study) random forest	Influence analysis for ordinal outcomes
Egger's Test	metabias6 lnor selnor	Publication bias test for ordinal outcomes
Funnel Plot	metafunnel lnor selnor	Funnel plot for ordinal outcomes
Trim and Fill Analysis	metatrim lnor selnor, funnel eform<br>metatrim lnor selnor, eform	Trim and fill analysis for ordinal outcomes (exponentiated results)

Dichotomous Variables Meta-Analysis

Analysis Objective	STATA Command	Description
Data Preparation	gen noevent_T = totalt - eventt	Calculate number of non-events

	gen noevent_C = totalc - eventc	for 2x2 table data
Risk Ratio Meta-Analysis	metan eventt noevent_T eventc noevent_C, rr	Meta-analysis of risk ratios using 2x2 table data
Influence Analysis	metaninf eventt noevent_T eventc noevent_C, id(study) random forest	Influence analysis for dichotomous outcomes

Implementation Notes:

Data Requirements: This code assumes the dataset is properly structured with the following variables: Continuous outcomes: n_exp, mean_exp, sd_exp, n_cotr, mean_cotr, sd_cotr Dichotomous outcomes: eventt, totalt, eventc, totalc Study identifiers: study

Package Dependencies: These commands require STATA meta-analysis packages including metan, metareg, metafunnel, and related modu

10 Supplementary Material S11. Detailed Adverse Events Across Included Studies

Study	Group	Specific adverse events (number of cases)	Overall incidence	Between-group comparison	Notes
Deng 2020	Basic (n=30)	Hypoglycemia 1, diarrhea 1	6.67% (2/30)	χ^2=0.74, P>0.05	Three-arm study; only T and C groups used in meta-analysis. All events mild.
Deng 2020	Control (n=30)	Hypoglycemia 1, diarrhea 1, hypotension 1, headache 1	13.33% (4/30)		
Deng 2020	Trial (n=30)	Hypoglycemia 1, diarrhea 1, headache 1	10.00% (3/30)		
Lou 2018	Both groups (n=90 each)	“Mild adverse reactions, did not affect treatment” – specific events not reported	Not reported	P>0.05	Qualitative description only.
Wang 2023	Both groups (n=44 each)	“No obvious adverse reactions occurred”	0%	—	No adverse events reported.
Su 2020	Control (n=47)	Flatulence 1, dizziness 2, headache 1, nausea 1	10.64% (5/47)	P<0.05	Significantly lower incidence in JLD group.
Su 2020	Observation (n=47)	Flatulence 1, nausea 1	4.26% (2/47)		

Study	Group	Specific adverse events (number of cases)	Overall incidence	Between-group comparison	Notes
Xia 2025	Observation (n=45)	Hypoglycemia 1, decreased appetite 2, pruritus 1	8.89% (4/45)	P>0.05	
Xia 2025	Control (n=45)	Hypoglycemia 1, decreased appetite 1	4.44% (2/45)		
Liu 2024	Control (n=47)	Dry mouth 1, fatigue 1, nausea 2	8.51% (4/47)	P>0.05	
Liu 2024	Observation (n=55)	Dry mouth 1, fatigue 1, nausea 1, rash 2	9.09% (5/55)		
Du 2012	Control (n=55)	Dyspepsia 1, mild diarrhea 2, postural hypotension 1	Not reported	Not reported	Gastric discomfort in treatment group resolved after postprandial administration.
Du 2012	Treatment (n=56)	Gastric discomfort 2 (resolved after postprandial administration)	Not reported		
Xu 2018	Trial (n=64)	Gastric discomfort 1 (resolved after postprandial administration)	Not reported	Not reported	Rash and postural hypotension in control resolved after symptomatic treatment.
Xu 2018	Control (n=64)	Postural hypotension 1, rash 2 (resolved after symptomatic treatment)	Not reported		
Ren 2019	Study (n=53)	Abdominal distension 1	1.89% (1/53)	P>0.05	Mild; resolved spontaneously.
Ren 2019	Control (n=53)	Nausea/vomiting 1	1.89% (1/53)		
Su 2017	Not specified	No information reported	—	—	Safety data unavailable.
Liu 2019	Treatment	Epigastric discomfort 1	Not reported	P>0.05	
Liu 2019	Control	Abdominal distension, decreased appetite 2	Not reported		
Chen 2019	Not specified	No information reported	—	—	Safety data unavailable.
